# Pathogenic variants in KCTD1 disrupt cAMP signaling and cellular communication associated with developmental pathways

**DOI:** 10.1016/j.jbc.2025.110813

**Published:** 2025-10-12

**Authors:** Yini Liao, Brian S. Muntean

**Affiliations:** Department of Pharmacology and Toxicology, Medical College of Georgia, Augusta University, Augusta, Georgia, USA

**Keywords:** potassium channel tetramerization domain 1 (KCTD1), G protein-coupled receptor (GPCR), cell signaling, signal transduction, cyclic AMP (cAMP), adenylate cyclase type 5 (AC5), KCTD, Wnt, Notch, Hh

## Abstract

The potassium channel tetramerization domain-containing 1 (KCTD1) protein has been associated with cancer, whereas pathogenic variants give rise to dental anomalies and aplasia cutis. KCTD1 has been implicated in signaling pathways connected to growth and development. Yet, the spectrum of pathology tied to KCTD1 underscores our nascent appreciation of its multifaceted roles. The objective of this study was to comprehensively examine the role of KCTD1 and its pathogenic variants across multiple signaling pathways in parallel. Here, we uncovered that KCTD1 human polymorphisms are more stable than previously suggested. Luminescence assays in cultured cells found that KCTD1 and its stable variants strongly inhibit Wnt, Hedgehog, and Notch pathways. We further identified that KCTD1 interacts with the Notch transcriptional repressor RBPJ. KCTD1 variants also shape G protein-coupled receptor (GPCR) signaling by increasing the abundance of adenylyl cyclase type 5 (AC5), which increases neuronal cAMP signaling in response to evoked dopamine in acute brain slices. Additionally, BRET-based biosensors revealed AC5 can compete with other effectors for Gβγ. Through this mechanism, KCTD1 is able to bias GPCR signals toward the cAMP pathway. Altogether, data demonstrate that KCTD1 is a general regulator of developmental (Wnt, Hedgehog, Notch) and GPCR (AC5→cAMP) signaling. Our study further establishes roles for KCTD1 variants across common signaling pathways.

Potassium channel tetramerization domain-containing (KCTD) proteins consist of 25 members hallmarked by a common bric-à-brac, tramtrack, broad complex (BTB) domain, which endows their ability to form interactions with numerous proteins (1, 2). KCTD1 stands out due to identification of multiple variants that give rise to dental anomalies (3) as well as scalp-ear-nipple syndrome (SEN; also known as Finlay–Marks syndrome), which is associated with aplasia cutis congenita (4). Many of the SEN point mutations occur in the BTB domain of KCTD1, including alanine 30, proline 31, histidine 33, glycine 62, and histidine 74 (4). The pathology of SEN suggests a role for KCTD1 and its critical BTB domain in cellular growth. Indeed KCTD1 has been implicated in cell differentiation through the Hedgehog (Hh) signaling pathway and development through the Wnt signaling pathway. Overexpression of KCTD1 in cells has been found to suppress Hh signaling (5); however, the impact of KCTD1 mutants has not been reported. KCTD1 knockout increases Wnt signaling in cell culture (6) and mouse distal convoluted tubules *in vivo* (7). While several KCTD1 mutants reportedly interfere with basal Wnt activity in cells (8), impact of variants on signaling driven by Wnt peptides remains unknown.

Several molecular mechanisms for KCTD1 have been documented. KCTD1 interaction with KCASH proteins suppresses Hh signaling (5). KCTD1 also binds and represses transactivation of the transcription factor TFAP2A (AP-2α) (9, 10), which is thought to play a role in regulating Wnt signaling (8, 11). The BTB domain enables assembly of multisubunit oligomers for most KCTD proteins (12, 13, 14). Hetero-oligomeric complex formation has also recently been observed for some KCTDs (15, 16, 17), including KCTD1 interaction with KCTD5 (18) and KCTD15 (19). KCTD1/KCTD15 complexes are important regulators of ectodermal and neural crest formation whereas the implications of KCTD1/KCTD5 have not yet been defined. KCTD5 impacts G protein-coupled receptor (GPCR) signaling to the cAMP cascade through modulation of the G protein subunit obligate dimer Gβγ (15, 20, 21, 22). KCTD1 is connected to GPCR signaling by stabilizing protein levels of the cAMP synthesizing enzyme adenylyl cyclase type 5 (AC5) (23). However interplay between KCTD1/AC5 with G proteins has not yet been examined.

The aim of our investigation was to elucidate the roles of KCTD1 in cellular communication by comprehensively comparing a series of signaling modalities. We report that most KCTD1 BTB mutations are very stable in transfected cells. Our data fortified previous work that KCTD1 negatively regulates Hh and Wnt signaling. In addition, stable KCTD1 BTB mutants suppress Hh and Wnt. Our study uncovered that KCTD1 also interacts with the transcriptional repressor recombination signal binding protein for immunoglobulin kappa J region (RBPJ) and inhibits the Notch signaling pathway. Similar to wildtype KCTD1, we found that stable BTB mutations upregulate AC5 to enhance cAMP signaling. Finally, we observed that increased AC5 level provides competition for Gβγ from other effectors, thus enabling KCTD1 to further bias signaling toward the cAMP pathway.

## Results

### Disease-causing KCTD1 mutations impact cellular stability

**Figure 1:**
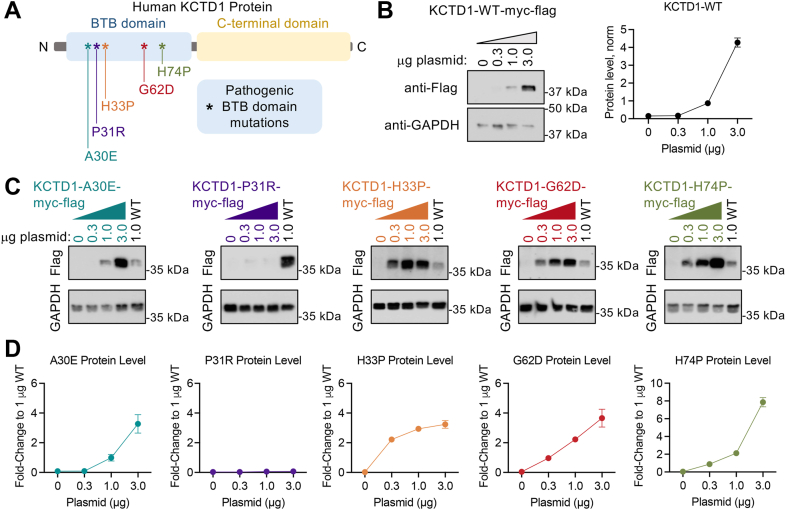
**Cellular stability of pathogenic BTB domain mutations in KCTD1.***A*, schematic of pathogenic mutations in human KCTD1 protein. *B*, Western blot of KCTD1 protein level in HEK293 cells at increasing plasmid transfections detected with an anti-Flag antibody. Representative blot from three experiments. *C*, Western blot of KCTD1 variants in HEK293 cells at increasing plasmid transfections with KCTD1-WT as a reference detected with an anti-Flag antibody. Representative blot from three experiments. *D*, Western blot quantification of KCTD1 mutants, normalized to 1.0 μg of KCTD1-WT. n = 3 independent experiments. BTB, bric-à-brac, tramtrack, broad complex; KCTD1, potassium channel tetramerization domain-containing 1; KCTD1-WT, wildtype KCTD1.

To gain insight into KCTD1 human polymorphisms, we first assessed protein stability in HEK293 cells. We cloned codon optimized KCTD1 containing pathogenic point mutations in the BTB domain (A30E, P31R, H33P, G62D, and H74P) into identical vectors with a CMV promoter and a C-terminal myc-flag epitope (Fig. 1*A*). HEK293 cells were transiently transfected, and Western blot was utilized to determine protein level with an anti-flag antibody. Wildtype KCTD1 (KCTD1-WT) was expressed in a dose-dependent manner with increasing amounts of plasmid transfection (0.3, 1.0, and 3.0 μg) (Fig. 1*B*). The same approach was applied to each KCTD1 variant while including KCTD1-WT (1.0 μg) as a reference (Fig. 1*C*). Each KCTD1 variant exhibited significantly increased protein level relative to KCTD1-WT except for P31R, which was undetected even at the highest plasmid transfection level (Fig. 1*D*). Data suggest that single amino acid substitutions in the KCTD1 BTB domain profoundly alter protein stability. Moreover, most pathogenic mutations in the BTB domain may significantly increase KCTD1 abundance.

### Impact of KCTD1 on developmental signaling

**Figure 2:**
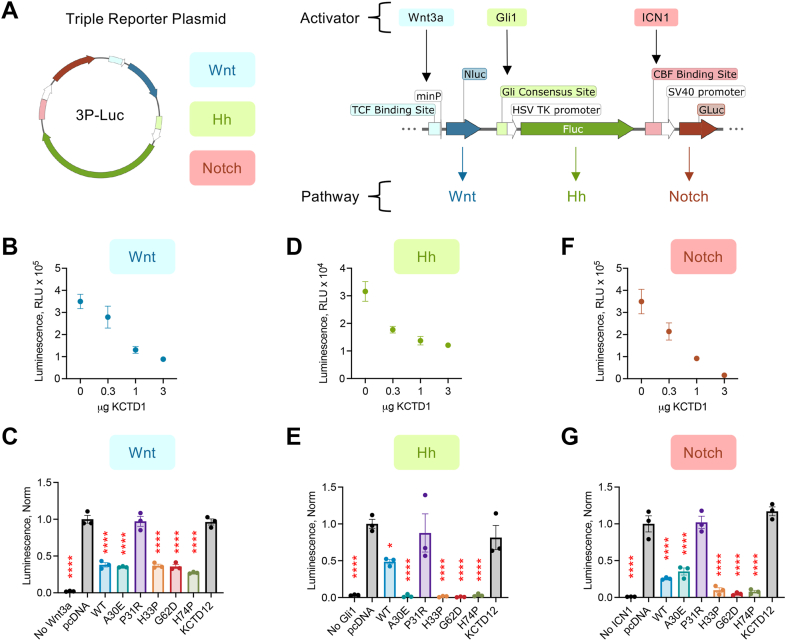
**Comparison of KCTD1 mutations on Wnt, Hh, and Notch signaling.***A*, schematic of 3P-Luc triple reporter luminescence assay. A single construct enables monitoring of Wnt (Nluc), Hh (Fluc), and Notch (Gluc) pathways. Wnt signaling was stimulated by treatment with Wnt3a peptide. Hh signaling was stimulated by cotransfection with Gli1. Notch signaling was stimulated by cotransfection with ICN1-GFP. *B*, luminescence quantification (Nano-Glo; Nluc) to HEK293 cells cotransfected with 3P-Luc, KCTD1, and pcDNA following overnight treatment with 100 ng/ml Wnt3a. n = 3 to 4 independent experiments. *C*, 3P-Luc Wnt response quantification in HEK293 cells cotransfected with KCTD1 variants (1.0 μg). n = 3 independent experiments. *D*, luminescence quantification (Bright-Glo; Fluc) to HEK293 cells cotransfected with 3P-Luc, Gli1, KCTD1, and pcDNA. n = 3 to 4 independent experiments. *E*, 3P-Luc Hh response quantification in HEK293 cells cotransfected with KCTD1 variants (1.0 μg). n = 3 independent experiments. *F*, luminescence quantification (Coelenterazine; Gluc) to HEK293 cells cotransfected with 3P-Luc, ICN1-GFP, KCTD1, and pcDNA. n = 4 independent experiments. *G*, 3P-Luc Notch response quantification in HEK293 cells cotransfected with KCTD1 variants (1.0 μg). n = 3 independent experiments. One-way ANOVA, Dunnett posttest, multiple comparison to pcDNA group; ∗ *p* < 0.05, ∗∗∗∗*p* < 0.0001. 3P-Luc, triple reporter plasmid; Fluc, Firefly luciferase; Gluc, Gaussia luciferase; Hh, Hedgehog; KCTD1, potassium channel tetramerization domain-containing 1; Nluc, Nanoluciferase.

KCTD1 has been mechanistically connected to both the Hh and Wnt signaling pathways (5, 8). However, disease causing mutations in KCTD1 have not been studied in Hh signaling while KCTD1’s cellular involvement in Wnt signaling has been controversial (24). Because of the developmental impact of KCTD1, we sought to resolve its role across the major developmental signaling pathways in parallel. We employed a luminescence approach with a triple reporter plasmid (3P-Luc) that encodes multiple genes to allow monitoring of Hh, Wnt, and Notch signaling (Fig. 2*A*) (25). 3P-Luc expresses three distinct luciferases: Nanoluciferase (Nluc), Firefly luciferase (Fluc), and Gaussia luciferase (Gluc). Each luciferase is driven by a promoter specific to the pathway of interest. Wnt induces Nluc expression (Nano-Glo substrate), Hh pathway induces Fluc (Bright-Glo substrate), and Notch signaling promotes Gluc (Coelenterazine substrate). Our first experiment gauged cross-talk between reporters and luciferase substrates (Fig. S1*A*). Under basal conditions (*i.e.,* no stimulation of respective pathways), the background luminescence was minimal from each luciferase. Overnight treatment with Wnt3a peptide (100 ng/ml) only induced luminescence in the Nluc channel. Transfection with Hh activator Gli1-flag only induced luminescence in the Fluc channel. Finally, transfection with the intracellular cleaved form of Notch1 only induced luminescence in the Gluc channel.

As an additional test of the 3P-Luc assay rigor, we repeated experiments with cotransfection of Gli1 (Hh activator; Fluc) and a cytosolic Nluc (Nluc-Cyto). The rationale is that data could be normalized if necessary to an internal control luminescence channel. We chose Nluc for this experiment because it is the brightest and most sensitive of the three luciferases encoded by 3P-Luc. In the absence of stimulation (*i.e.,* only 3P-Luc and pcDNA transfection), there was minimal luminescence signal (Fig. S1*B*). As before, 3P-Luc and Gli1 produced luminescence exclusively in the Fluc channel. Cotransfection of 3P-Luc with Nluc-Cyto induced robust luminescence exclusively from the Nluc substrate. This confirmed expression of Nluc-Cyto on top of the Nluc from the 3P-Luc vector. Finally, transfection of 3P-Luc along with Gli1 and Nluc-Cyto induced luminescence in both Nluc and Fluc readouts, which were of similar values from experiments with either Gli1 or Nluc-Cyto alone. In each of these experiments, there was minimal signal, as expected, from the Gluc substrate. This data collectively suggests compatibility of internal control luminescence vectors for data normalization and ultimately confirm utility of the 3P-Luc system to parse signaling in each respective pathway.

Wnt signaling was then examined in 3P-Luc–transfected cells by overnight treatment of Wnt3a peptide (100 ng/ml) followed by measuring the luminescence signal from the Nluc reporter. Cotransfection of KCTD1-WT significantly reduced Wnt signaling in a dose-dependent manner (Fig. 2*B*). Each KCTD1 BTB domain mutant was then tested (1.0 μg transfection amount), which reduced Wnt signaling to approximately the same degree as KCTD1-WT (at 1.0 μg transfection) (Fig. 2*C*). The notable exception was that P31R had no effect, and as a result, Wnt signaling mirrored the activity observed in the control transfection (pcDNA empty vector at 1.0 μg). As an additional control, the relatively well-studied KCTD12 was also examined as it participates in distinct cellular processes compared with KCTD1 (17, 26). As anticipated, KCTD12 did not impact Wnt signaling (Fig. 2*C*). The major benefit of the 3P-Luc approach is an all-in-one system to screen pathway alterations. Nonetheless, a potential caveat is reliance on absolute luminescence subject to nonspecific factors, including transfection efficiency, changes in transcription factor expression, or cell viability. Hence, we fortified our results by repeating the assay while cotransfecting a cytosolic Fluc (Fig. S1*C*). The raw Wnt signal readout (Nluc) was then normalized to raw Fluc values (Fig. S1*D*). We observed KCTD1 and its variants, except for P31R, significantly reduced Wnt signaling in comparison with pcDNA control (Fig. S1*E*).

**Figure 3:**
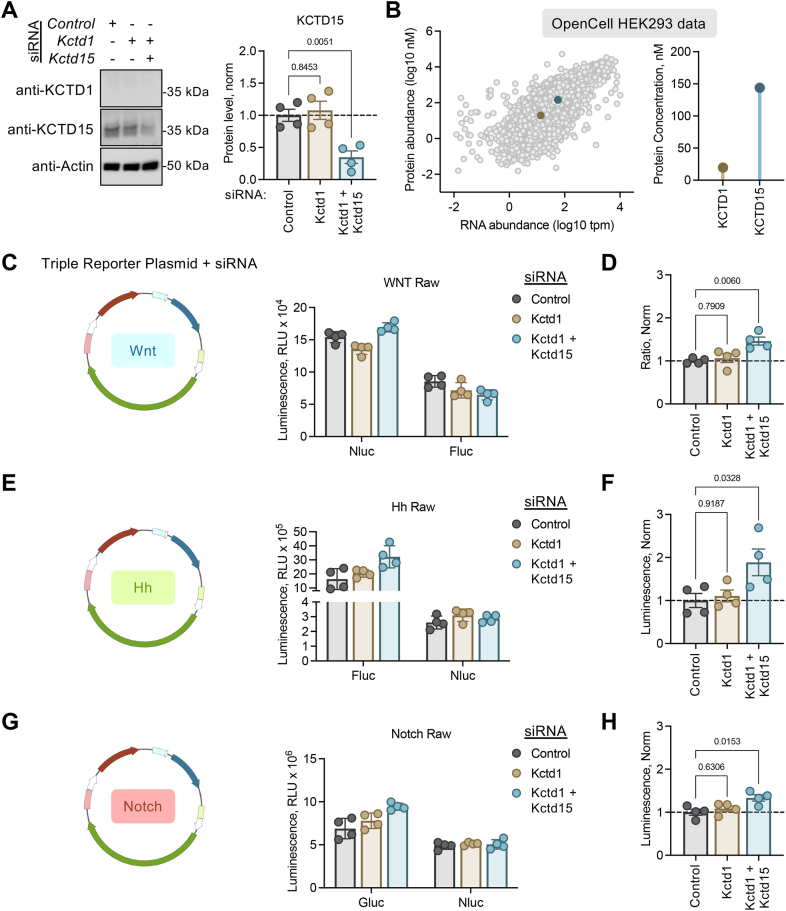
***Kctd15* knockdown increases Wnt, Hh, and Notch signaling.***A*, Western blot analysis of KCTD1 and KCTD15 level in HEK293 cells transfected with control, *Kctd1*, or *Kctd1* and *Kctd15* siRNA. KCTD1 was not detected, and KCTD15 protein quantification was normalized to control siRNA group. n = 4 independent experiments. One-way ANOVA, Dunnett posttest, multiple comparison to 0 μg KCTD1-mVenus group; exact *p* values indicated on the bar graph. *B*, abundance of KCTD1 and KCTD15 in HEK293 cells. Data curated from OpenCell database (27). *C*, luminescence quantification of 3P-Luc assay to examine Wnt (Nluc) signaling, with cytosolic Fluc cotransfection to normalize data, in siRNA-transfected HEK293 cells following overnight treatment with 100 ng/ml Wnt3a. n = 4 independent experiments. *D*, quantification of luminescence ratio (Nluc Wnt signal divided by Fluc internal control) normalized to control siRNA group. n = 4 independent experiments. One-way ANOVA, Dunnett posttest, and multiple comparison to control siRNA group; exact *p* values indicated on the bar graph. *E*, luminescence quantification of 3P-Luc assay to examine Hh (Fluc) signaling, with cytosolic Nluc cotransfection to normalize data, in siRNA transfected HEK293 cells. n = 4 independent experiments. *F*, quantification of luminescence ratio (Fluc Wnt signal divided by Nluc internal control) normalized to control siRNA group. n = 4 independent experiments. One-way ANOVA, Dunnett posttest, and multiple comparison to control siRNA group; exact *p* values indicated on the bar graph. *G*, luminescence quantification of 3P-Luc assay to examine Notch (Gluc) signaling, with cytosolic Nluc cotransfection to normalize data, in siRNA transfected HEK293 cells. n = 4 independent experiments. *H*, quantification of luminescence ratio (Gluc Wnt signal divided by Nluc internal control) normalized to control siRNA group. n = 4 independent experiments. One-way ANOVA, Dunnett posttest, and multiple comparison to control siRNA group; exact *p* values indicated on the bar graph. Fluc, Firefly luciferase; Hh, Hedgehog; Nluc, Nanoluciferase.

The Hh pathway was next examined by cotransfection of HEK293 cells with 3P-Luc and the Hh activator Gli1, which generated a robust luminescence signal from the Fluc reporter (Fig. 2*D*). Transfection of KCTD1 induced a dose-dependent decrease in luminescence (Fig. 2*D*), confirming that KCTD1 suppresses Hh signaling. We subsequently examined the KCTD1 variants (1.0 μg transfection). Similar to before, P31R had no impact on Hh (Fig. 2*E*). The other KCTD1 variants completely blunted Hh signaling, which is striking given that KCTD1-WT only reduced the luminescence response by roughly 50%. The KCTD12 control had no effect on Hh signaling (Fig. 3*E*). We then repeated the experiments with cotransfection of Nluc-Cyto serving as an internal normalization factor (Fig. S2, *A* and *B*). This resulted in a significant reduction of Hh signaling by KCTD1 and its variants, except for P31R, compared to pcDNA control (Fig. S2*C*). To assess if KCTD1 potentially interfered with Gli1 stability, we used Western blot to directly measure Gli1 level while increasing concentrations of KCTD1 (Fig. S2*D*). However, there were no significant changes in Gli1 level suggesting that results in the reporter assay were downstream and not due to suppressed Gli1 (Fig. S3*E*).

Finally, Notch signaling was examined by cotransfection of 3P-Luc with ICN1, which drives transcription of Gluc through the CBF promoter. Cotransfection of KCTD1 significantly reduced Notch signaling in a dose-dependent manner (Fig. 2*F*). The KCTD12 control and P31R variant had no effect on signaling (Fig. 2*G*). The A30E variant reduced Notch signaling by the same amount as KCTD1-WT (all at 1.0 μg). Yet, the Notch response was even further reduced with near complete inhibition by H33P, G62D, and H74P (Fig. 2*G*). We again validated the assay results by performing experiments with cotransfection of cytosolic Nluc for normalization (Fig. S3, *A* and *B*). We observed the same trend whereby KCTD1 and its stable variants significantly suppressed Notch signaling (Fig. S3*C*). Assessment of ICN1 level was then measured by Western blot during cotransfection of increasing amounts of KCTD1 (Fig. S3*D*); however, KCTD1 did not impact ICN1 level (Fig. S3*E*).

If overexpression of KCTD1 reduced signaling, it is conceivable that lowering KCTD1 level may result in elevated signaling. We attempted to address this question by using siRNA to knockdown KCTD1 in HEK293 cells. Despite using a validated antibody (23), we were unable to detect endogenous KCTD1 in HEK293 cells (Fig. 3*A*). KCTD1 is very similar to KCTD15 (80% identity), and the two proteins have been found to exhibit overlapping roles in neural crest cell formation (19). Endogenous KCTD1 is relatively low in HEK293 cells while KCTD15 is far more abundant at both the RNA and protein level (27) (Fig. 3*B*). Therefore, we tested the hypothesis that in HEK293 cells KCTD15 knockdown may elevate the signaling modalities regulated by KCTD1 overexpression. Our experiments accounted for potential compensation utilizing (i) scrambled siRNA control, (ii) *Kctd1*-targeted siRNA, (iii) *Kctd1*-targeted siRNA, and (iv) *Kctd15*-targeted siRNA. We first confirmed detection of endogenous KCTD15 (Fig. 3*A*) and then found that *Kctd1/Kctd15*-targeted siRNA was able to significantly reduce KCTD15 protein level compared with control (Fig. 3*A*). The siRNA strategy was next incorporated into 3P-Luc assays. For the Wnt pathway, cytosolic Fluc was cotransfected for an internal normalization control (Fig. 3*C*). We found the Wnt3a treatment resulted in a significantly greater Wnt response in *Kctd1/Kctd15*-targeted siRNA cells compared with control (Fig. 3*D*). Similarly in the Hh pathway, where cotransfected cytosolic Nluc was used for data normalization (Fig. 3*E*), loss of KCTD15 significantly increased the Gli1-induced response (Fig. 3*F*). Finally, the Notch reporter cotransfected with cytosolic Nluc (Fig. 3*G*) also led to an ICN1-induced response that was significantly elevated during KCTD15 knockdown (Fig. 3*H*). The results indicate that loss of KCTD15 (a redundant isoform of KCTD1) leads to increased signaling in the Wnt, Hh, and Notch pathways.

Collectively, our data suggest KCTD1 is a negative regulator of three important developmental signaling pathways. Furthermore, BTB-domain point mutations in KCTD1 may play a role in signal regulation with the exception of P31R, most likely due to its instability in transfected HEK293 cells.

### KCTD1 overexpression suppresses smoothened agonist–induced Hh signaling in HEK293 and NIH/3T3 cell lines

**Figure 4:**
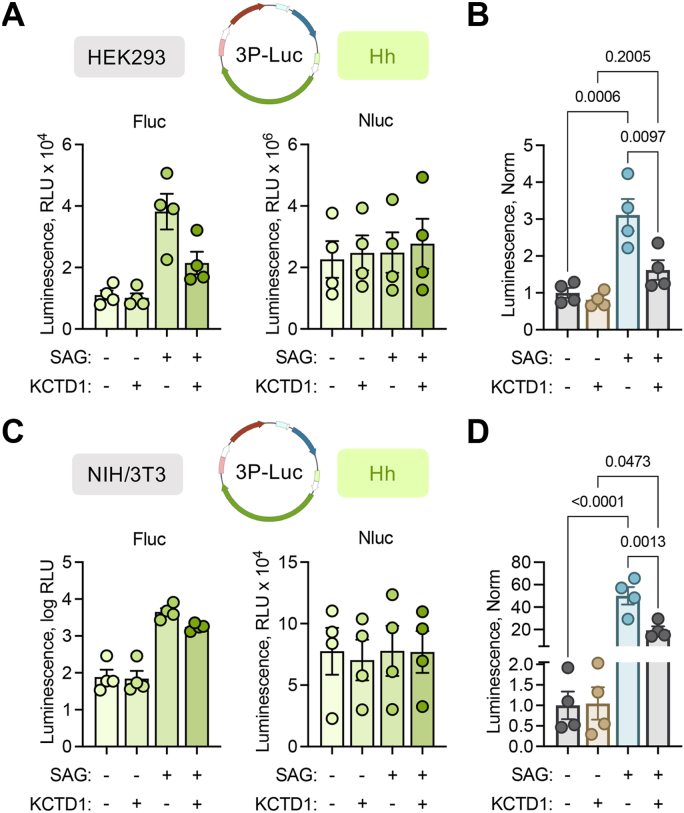
**KCTD1 overexpression reduces SAG-induced Hh signaling.***A*, luminescence quantification of 3P-Luc assay to examine Hh (Fluc) signaling, with cytosolic Nluc cotransfection to normalize data, in SAG (2.5 μM) treated HEK293 cells. n = 4 independent experiments. *B*, quantification of luminescence ratio (Fluc Hh signal divided by Nluc internal control) normalized to control group (0 SAG, 0 KCTD1) in HEK293 cells. n = 4 independent experiments. One-way ANOVA, Dunnett posttest, and multiple comparison to control group; exact *p* values indicated on the bar graph. *C*, luminescence quantification of 3P-Luc assay to examine Hh (Fluc) signaling, with cytosolic Nluc cotransfection to normalize data, in SAG (2.5 μM) treated NIH/3T3 cells. n = 4 independent experiments. *D*, quantification of luminescence ratio (Fluc Hh signal divided by Nluc internal control) normalized to control group (0 SAG, 0 KCTD1) in NIH/3T3 cells. n = 4 independent experiments. One-way ANOVA, Dunnett posttest, and multiple comparison to control group; exact *p* values indicated on the bar graph. Fluc, Firefly luciferase; Hh, Hedgehog; KCTD1, potassium channel tetramerization domain-containing 1; Nluc, Nanoluciferase; SAG, smoothened agonist.

To better understand physiological implications of KCTD1 in the Hh pathway, we next treated 3P-Luc-transfected HEK293 cells with the smoothened agonist (SAG; 2.5 μM) to stimulate the endogenous signaling cascade. Cytosolic Nluc was used to normalize data, and cells were cotransfected with either KCTD1 or pcDNA (Fig. 4*A*). SAG induced a 3-fold increase in Hh signaling compared to vehicle in the pcDNA group, whereas overexpression of KCTD1 reduced the SAG response by about 50% compared with pcDNA (Fig. 4*B*). As HEK293 cells do not efficiently form primary cilia, which is important for Hh signaling, we next fortified our findings with the NIH/3T3 cell line that is more suitable for Hh (28) and cilia-dependent processes (29, 30). We started with the 3P-Luc assay in NIH/3T3 cells with normalization to cytosolic Nluc (Fig. S4*A*). Transfection of Gli1 robustly increased the luminescence readout, which was significantly reduced with cotransfection of KCTD1 (Fig. S4*B*). We next investigated the effect of SAG on NIH/3T3 cells in the presence or absence of KCTD1 (Fig. 4*C*). SAG increased Hh signaling, and the effect was significantly reduced in the presence of KCTD1 (Fig. 4*D*). These data suggest KCTD1 may reduce Hh signaling even in nonciliated cells whether stimulated by Gli1 transfection or SAG treatment.

### KCTD1 inhibits Notch signaling and interacts with RBPJ

**Figure 5:**
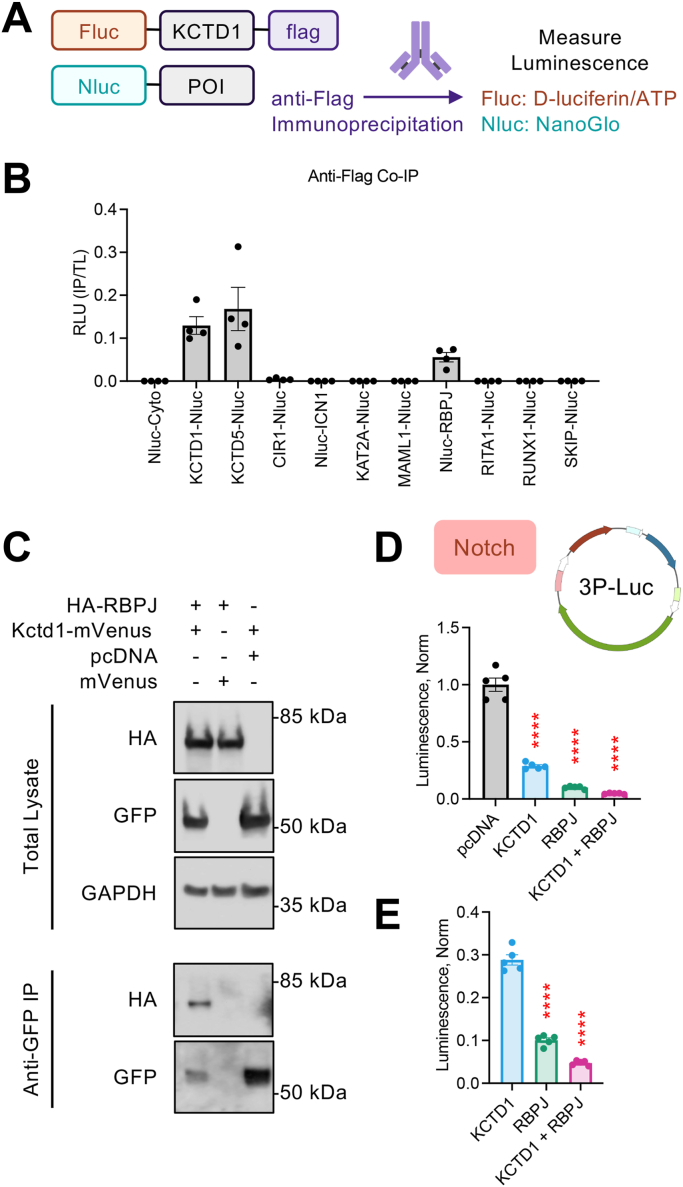
**KCTD1 interacts with RBPJ and regulates Notch signaling.***A*, schematic for IP luminescence strategy for HEK293 cells cotransfected with Fluc-KCTD1-flag and Nluc-tagged proteins of interest (POI) in the Notch signaling cascade. *B*, quantification of Nluc luminescence ratio (IP/total lysate) from HEK293 cells cotransfected with Fluc-KCTD1-flag and Nluc-tagged POI. n = 4 independent experiments. *C*, IP of KCTD1-mVenus from HEK293 cells with an anti-GFP antibody followed by Western blot for HA-RBPJ with an anti-HA antibody. Representative blot from three independent experiments. *D*, Notch response quantification (Coelenterazine; Gluc) in HEK293 cotransfected with 3P-Luc and ICN1-GFP along with pcDNA, KCTD1-WT, and RBPJ as indicated. n = 5 independent experiments. One-way ANOVA, Dunnett posttest, and multiple comparison to pcDNA group; ∗∗∗∗*p* < 0.0001. *E*, same data as panel 3C with omission of pcDNA group (n = 5 independent experiments). One-way ANOVA, Dunnett posttest, multiple comparison to KCTD1 group; ∗∗∗∗*p* < 0.0001. Fluc, Firefly luciferase; KCTD1, potassium channel tetramerization domain-containing 1; KCTD1-WT, wildtype KCTD1; RBPJ, repressor recombination signal binding protein for immunoglobulin kappa J region.

We next pursued mechanistic insight toward how Notch signaling is regulated by KCTD1. Notch signaling does not utilize second messengers rather the transmembrane receptor is cleaved to ICN1 (31). Nuclear translocation of ICN1 leads to interaction with a multitude of transcription regulators that modulate signaling output (32). The 3P-Luc assay utilized ICN1, suggesting KCTD1 intervention took place downstream of Notch receptor activation. Therefore, we employed confocal microscopy to examine if ICN1 nuclear localization was affected by KCTD1. We fused mVenus to the C terminus of ICN1 and cotransfected HEK293 cells with membrane (mCherry-KRas) and nuclear (3X-NLS-mTurquoise2) markers in the presence of increasing amounts of KCTD1 (Fig. S5*A*). Regardless of the KCTD1 level, ICN1 localization remained in the nucleus (Fig. S5, *B* and *C*), suggesting KCTD1 may interfere with the functional role of ICN1. We were unable to identify an interaction between KCTD1 and ICN1 by co-immunoprecipitation (Co-IP) (*Data not shown*). This prompted investigation into interaction between KCTD1 and the regulators of Notch signaling. We selected eight key genes previously reported to be engaged with ICN1 and fused their cDNA with Nluc. Each Nluc-tagged protein of interest was then cotransfected with Fluc-KCTD1-flag in HEK293 cells for Co-IP with an anti-flag antibody followed by dual luminescence measurements (Fig. 5*A*). Quantification of Fluc (KCTD1) and Nluc (Notch protein of interest) in total cell lysates were roughly equivalent, suggesting each construct expressed at similar levels (Fig. S6, *A* and *B*). Of note is that most Nluc-tagged proteins exhibited significantly reduced luminescence compared with the control Nluc-Cyto, which is likely due to the extreme stability of untethered Nluc. Nonetheless, each Nluc-tagged protein was strongly expressed. Fluc in IP samples was also consistent, indicating robust KCTD1 pulldown across experiments (Fig. S6*C*). As anticipated in the negative control, signal from Nluc-Cyto was absent from IP samples (Fig. S6*D*). KCTD1 has been shown to form homo-oligomers as well as hetero-oligomers with KCTD5 (18), and we indeed observed luminescence from KCTD1-Nluc and KCTD5-Nluc in the IP samples (Fig. S6*D*). Interestingly, the Notch repressor RBPJ stood out for its robust luminescence in the IP samples (Fig. S6*D*). Indeed ratiometric analysis of Nluc (IP/TL) suggested that among the eight genes selected, only RBPJ exhibited an interaction with KCTD1 (Fig. 5*B*). We next examined if KCTD1 interacted with RBPJ in HEK293 cells transfected with HA-RBPJ and KCTD1-mVenus. Co-IP was performed with an anti-GFP antibody followed by Western blot. Indeed, we observed a robust interaction between KCTD1 and RBPJ (Fig. 5*C*). To test the functional consequences, we returned to the 3P-Luc assay. As demonstrated in Figure 2, KCTD1 significantly blunted Notch signaling compared with control (pcDNA empty vector) (Fig. 5*D*). The RBPJ repressor also significantly reduced Notch signaling. Cotransfection of both KCTD1 and RBPJ had a synergistic effect on significantly lowering Notch signaling relative to KCTD1 or RBPJ alone (Fig. 5*E*). These data suggest that KCTD1 mechanistically restricts Notch signaling and interacts with RBPJ.

### Disease-causing KCTD1 variants impact on AC5 and cAMP

**Figure 6:**
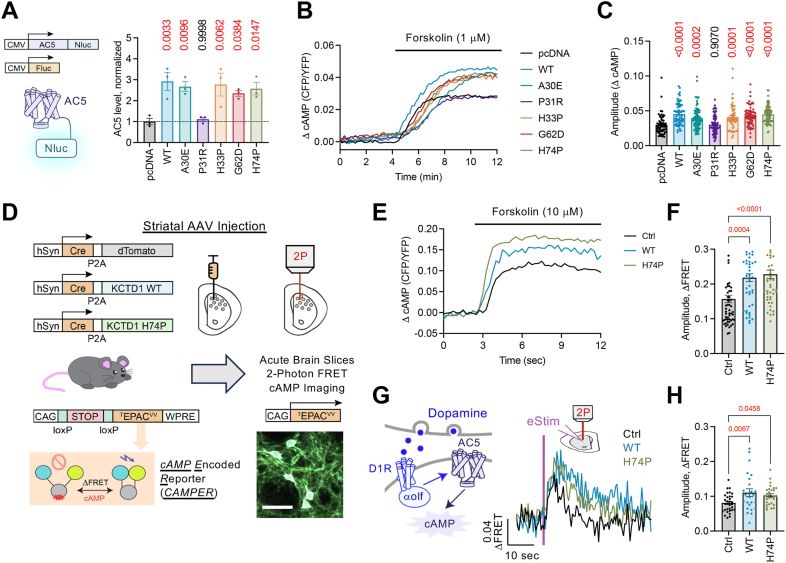
**KCTD1 regulation of cAMP signaling in cells and striatal neurons.***A*, quantification of AC5-Nluc level, normalized to Fluc, in HEK 293 cells cotransfected with either pcDNA or KCTD1 (1 μg). n = 3 independent experiments. One-way ANOVA, Dunnett posttest, and multiple comparison to pcDNA group. *B*, representative cAMP response to 1 μM forskolin in HEK 293 cells cotransfected with ^T^Epac^VV^, flag-AC5, and either pcDNA or KCTD1 (1 μg). *C*, maximum forskolin (1 μM) induced cAMP amplitude in HEK 293 cells. n = 4 independent experiments with the following total number of cells analyzed: pcDNA (64), KCTD1-WT (64), A30E (86), P31R (72), H33P (57), G62D (71), and H74P (70). One-way ANOVA, Dunnett posttest, and multiple comparison to pcDNA group. *D*, schematic of stereotaxic injection of AAV particles encoding Cre and KCTD1 or dTomato in the dorsal striatum of CAMPER mice for 2-photon imaging in acute brain slices (representative fluorescence from 300 μm acute slice; white scale bar represents 50 μm). *E*, average trace of cAMP responses to 10 μM forskolin from CAMPER striatal neurons in acute brain slices: Control (58 neurons/5 animals), KCTD1-WT (47 neurons/6 animals), and KCTD1-H74P (49 neurons/6 animals). *F*, maximum forskolin (10 μM) induced cAMP amplitude from CAMPER striatal neurons in acute brain slices: Control (58 neurons/5 animals), KCTD1-WT (47 neurons/6 animals), and KCTD1-H74P (49 neurons/6 animals). One-way ANOVA, Dunnett posttest, and multiple comparison to Control group. *G*, representative stimulatory trace to electrically evoked dopamine from CAMPER striatal neurons in acute brain slices: Control (32 neurons/5 animals), KCTD1-WT (25 neurons/6 animals), and KCTD1-H74P (30 neurons/6 animals). *H*, maximum stimulatory cAMP response to electrically evoked dopamine from CAMPER striatal neurons in acute brain slices: Control (32 neurons/5 animals), KCTD1-WT (25 neurons/6 animals), and KCTD1-H74P (30 neurons/6 animals). One-way ANOVA, Dunnett posttest, and multiple comparison to control group. AC5, adenylyl cyclase type 5; *CAMPER, cAMP e*ncoded *r*eporter; KCTD1, potassium channel tetramerization domain-containing 1; KCTD1-WT, wildtype KCTD1; Nluc, Nanoluciferase.

We previously found KCTD1 promotes de-glycosylation of AC5, which in turn prevents AC5 ubiquitination and promotes AC5 abundance. Therefore we wanted to examine if KCTD1 variants may similarly regulate AC5 level. HEK293 cells were cotransfected with AC5-Nluc, Fluc, and KCTD1-myc-flag. The luminescence ratio of Nluc to Fluc was calculated to provide a readout for AC5 level (Fig. S7*A*). We found that wildtype and KCTD1 variants significantly increased AC5 level, with the predicted exception of P31R (Fig. 6*A*). To determine functional consequences of AC5 upregulation, we optically recorded cAMP signaling using a FRET-based cAMP biosensor (^T^Epac^VV^) (33) (Fig. S7*B*). HEK293 cells were cotransfected with the biosensor and flag-AC5 in the presence or absence of KCTD1. Stimulation with the AC-activator forskolin (1 μM) triggered robust production of cAMP that was significantly augmented by overexpression of KCTD1 (Figs. 6, *B*, *C* and S8). In the same or similar fashion, each KCTD1 variant (A30E, H33P, G62D, and H74P) enhanced forskolin responses relative to pcDNA control (Figs. 6, *B*, *C* and S8). As anticipated from previous data, the KCTD1 variant P31R did not impact forskolin efficacy. These data support the notion that KCTD1 enhances functional level of AC5 in cells. Although the diterpene natural product forskolin has been well-appreciated to gauge AC activity (34), we still wanted to examine in a more physiological context. Thus, we turned our attention to striatal neurons, which are thought to rely on AC5 for ∼80% of cAMP signaling (35). For neuronal expression, we packaged the KCTD1 ORF (or dTomato control) into an AAV vector that also encodes Cre (AAV-Cre-P2A-KCTD1-WT and AAV-Cre-P2A-dTomato). We studied the H74P mutation as a representative variant as it demonstrated a robust effect on AC5 in the previous data (AAV-Cre-P2A-KCTD1-H74P). AAV particles were then stereotactically injected into the striatum of *cAMP e*ncoded *r*eporter (*CAMPER*) mice, which conditionally express the ^T^Epac^VV^ biosensor (Fig. 6*D*) (36). Acute brain slices were then prepared to record cAMP changes from dorsal striatum with 2-photon confocal microscopy. Bath application of forskolin induced significantly greater cAMP responses in KCTD1-WT or KCTD1-H74P overexpressing cells compared with control (dTomato) (Fig. 6, *E* and *F*). Because striatal neurons decode dopamine transients for many physiological responses (37), we used a stimulating electrode to evoke dopamine release while simultaneously recording cAMP changes (Fig. 6*G*). Striatal neurons are segregated relative to their expression of dopamine 1 receptor (D1R; stimulates cAMP) and dopamine 2 receptor (D2R; inhibits cAMP). Our analysis focused on stimulatory signaling and therefore only considered neuronal responses in the positive direction, as previously performed (20, 23, 38). In these experiments, overexpression of KCTD1-WT or KCTD1-H74P resulted in significantly enhanced D1R→cAMP responses relative to control (Fig. 6*H*). Remarkably, measurements of cAMP following synaptically evoked dopamine yielded an approximate 30% increase during KCTD1 overexpression. Such strong influence on endogenous dopamine signaling provides further support for motor behavior alterations observed in striatal *Kctd1* knockdown (23). Altogether, our data suggest that KCTD1 and its stable BTB-domain variants enhance cAMP signaling by increasing the abundance of AC5 protein level.

### AC5 competes with effectors for Gβγ, which is facilitated by KCTD1

**Figure 7:**
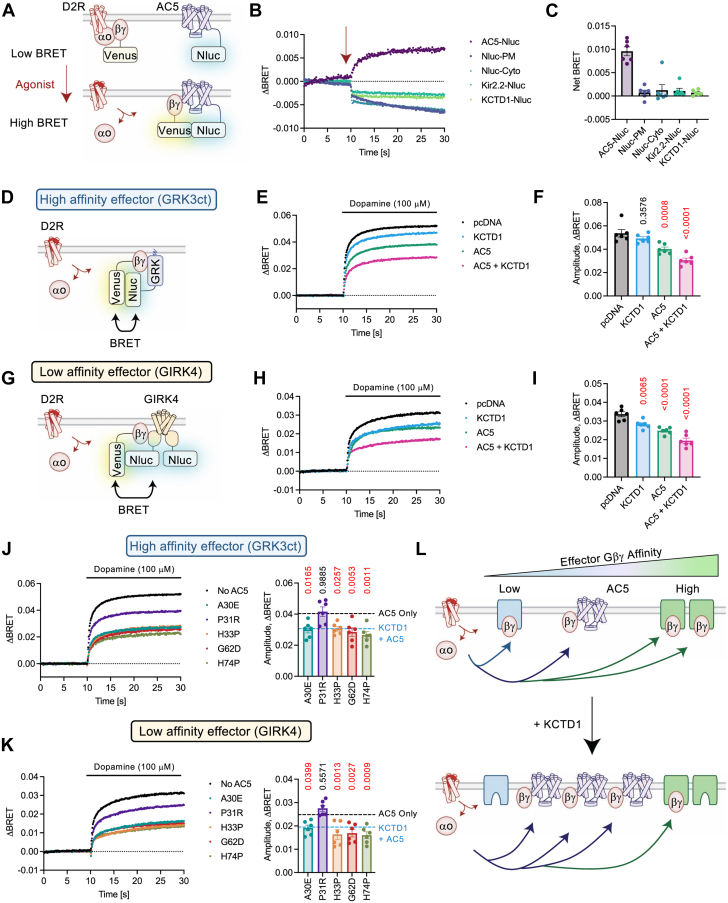
**KCTD1 and AC5 provide competition for Gβγ effectors.***A*, schematic of BRET assay for dopamine-D2R mediated association of Gβγ-Venus with AC5-Nluc. *B*, average BRET trace induced by 100 μM dopamine from HEK293 cells cotransfected with D2R, Gαo, Gβγ-Venus, and indicated Nluc-tagged protein. n = 6 independent experiments. *C*, quantification of BRET amplitude from 100 μM dopamine. n = 6 independent experiments. *D*, schematic of BRET assay for dopamine-D2R mediated association of Gβγ-Venus with GRK3ct-Nluc. *E*, average BRET trace induced by 100 μM dopamine from HEK293 cells cotransfected with D2R, Gαo, Gβγ-Venus, and GRK3ct-Nluc. n = 6 independent experiments. *F*, quantification of BRET amplitude from 100 μM dopamine. n = 6 independent experiments. One-way ANOVA, Dunnett posttest, and multiple comparison to pcDNA group. *G*, schematic of BRET assay for dopamine-D2R mediated association of Gβγ-Venus with GIRK4-Nluc. *H*, average BRET trace induced by 100 μM dopamine from HEK293 cells cotransfected with D2R, Gαo, Gβγ-Venus, and GIRK4-Nluc. n = 6 independent experiments. *I*, quantification of BRET amplitude from 100 μM dopamine. n = 6 independent experiments. One-way ANOVA, Dunnett posttest, and multiple comparison to pcDNA group. *J*, average BRET trace and amplitude quantification induced by 100 μM dopamine from HEK293 cells cotransfected with D2R, Gαo, Gβγ-Venus, GRK3ct-Nluc, and indicated KCTD1. n = 6 independent experiments. One-way ANOVA, Dunnett posttest, and multiple comparison to AC5 group. *Blackdotted line* indicates amplitude from AC5 transfection (AC5 Only), and *blue dotted line* indicates amplitude from cotransfection of AC5 and KCTD1. *K*, average BRET trace and amplitude quantification induced by 100 μM dopamine from HEK293 cells cotransfected with D2R, Gαo, Gβγ-Venus, GIRK4-Nluc, and indicated KCTD1. n = 6 independent experiments. One-way ANOVA, Dunnett posttest, and multiple comparison to pcDNA group. *Blackdotted line* indicates amplitude from AC5 transfection (AC5 Only), and *blue dotted line* indicates amplitude from cotransfection of AC5 and KCTD1. *L*, schematic of Gβγ-effector bias provided by KCTD1-mediated increase of AC5 abundance. AC5, adenylyl cyclase type 5; D2R, dopamine 2 receptor; KCTD1, potassium channel tetramerization domain-containing 1; Nluc, Nanoluciferase.

KCTD1 mutations have not been linked to striatal associated pathology suggesting additional nodes of signaling could be altered. While other mechanisms of action for KCTD1 have been described (39), KCTD1-mediated regulation of AC5 strongly suggests its importance (23). Therefore, we considered the possibility that AC5 may provide crosstalk to adjacent signaling modalities. Our lab and others have established that AC5 activity is sensitized by Gβγ binding (40, 41, 42, 43). Because KCTD1 increases AC5 abundance (23) and mobilized G proteins are routed throughout the cell (44, 45, 46), we hypothesized that excess AC5 could scavenge Gβγ from other effectors. We first investigated if AC5 could interact with Gβγ following GPCR stimulation in a BRET-based assay in live cells. Here, we transfected HEK293 cells with AC5 fused to Nanoluciferase (AC5-Nluc), D2R, GαoA, and Gβγ-Venus (Venus156-239-Gβ1 and Venus1-155-Gγ2) (Fig. 7*A*). A saturating concentration of dopamine induced a robust BRET signal between AC5-Nluc and Gβγ-Venus, indicating interaction on a timeframe compatible with GPCR signaling (Fig. 7*B*). For negative controls, we utilized the C-terminal plasma membrane targeting region of KRas fused to Nluc (Nluc-PM), a cytosolic Nanoluciferase (Nluc-Cyto), and an ion channel that does not bind Gβγ (Kir2.2-Nluc). We also checked if KCTD1 would interact with Gβγ following GPCR stimulation (KCTD1-Nluc). Each negative control failed to generate a BRET signal in response to dopamine (Fig. 7, *B* and *C*). As previously reported (21), KCTD1 did not interact with Gβγ following receptor stimulation, thus further validating the results. We additionally tested if KCTD1 interferes with G protein activation. The BRET assay was performed as classically described with D2R, Gαo, Gβγ-Venus, and GRK3ct-Nluc (47, 48) but in the presence or absence of KCTD1. There was no impact of KCTD1 on G protein activation from D2R→Gαo (Fig. S9*A*). Signaling through D1R→Gαs (Fig. S9*A*) and M3R→Gαq (Fig. S9*C*) was similarly assessed; however, KCTD1 had no impact on G protein activation. Data suggest KCTD1 does not seem to directly influence G protein activation.

This allowed utilization of the BRET approach to study if AC5 could scavenge Gβγ from either a high (GRK3ct: ∼nM) or low (GIRK4: ∼mM) affinity effector fused to Nluc (GRK3ct-Nluc and GIRK4-Nluc) (49, 50). For positive controls, we utilized GRK3ct without the Nluc tag in addition to the previously characterized Gβγ-binding nanobody (Nanobody 5; Nb5) (51). For a negative control, we used the matching nanobody that exhibits indifference toward Gβγ (Nanobody 17; Nb17) (51). Beginning with GRK3ct-Nluc, D2R stimulation induced a robust BRET response that was significantly reduced in the presence of Gβγ scavengers (GRK3ct and Nb5), whereas Nb17 elicited no effect (Fig. S10, *A* and *B*). Similarly, the agonist-induced BRET between Gβγ-Venus and GIRK4-Nluc was significantly diminished by either GRK3ct or Nb5 but not Nb17 (Fig. S10, *C* and *D*). We next looked into how AC5 would impact BRET response between Gβγ-Venus and GRK3ct (Fig. 7*D*). Overexpression of wildtype AC5 significantly reduced BRET amplitude, whereas the signal was even further dampened in the presence of both KCTD1 and AC5 (Fig. 7, *E* and *F*). As KCTD1 alone did not impact BRET, data suggest increased abundance of AC5 was sufficient to sequester Gβγ from GRK3ct (Fig. 7, *E* and *F*). Similarly, presence of AC5 reduced the agonist-induced BRET signal between GIRK4-Nluc and Gβγ-Venus (Fig. 7, *G*–*I*). Additionally, the combination of KCTD1 with AC5 even further reduced the GIRK4–Gβγ interaction. Based on our findings that some KCTD1 variants (A30E, H33P, G62D, H74P) strongly promote AC5, we next examined how these mutants might impact Gβγ interaction with effectors. As anticipated, cotransfection of these variants with AC5 significantly reduced agonist-induced BRET responses between Gβγ with either GRK3ct (Fig. 7*J*) or GIRK4 (Fig. 7*K*). We also observed the unstable variant, P31R, had no impact on sequestering Gβγ. Altogether, our data support a model that AC5 competes with effectors for Gβγ. By increasing AC5 abundance, KCTD1 provides a mechanism for GPCR crosstalk to bias signals at the effector level (Fig. 7*L*).

## Discussion

We highlight several key aspects of KCTD1’s cellular functions. The major findings solidify the role of KCTD1 and its pathogenic mutants in developmental (Wnt, Hh, and Notch) and G protein signaling pathways. We identify a novel binding partner for KCTD1 in the Notch repressor RBPJ. The results also present a mode of signal bias where KCTD1’s upregulation of AC5 enables competition with effectors for Gβγ.

We first observed that most pathogenic mutations in KCTD1 result in enhanced protein abundance. A30E, H33P, and G62D were approximately 3.5-fold more stable in transfected cells compared to WT, whereas H74P was roughly 8-fold more abundant. The only exception was P31R, which was completely undetectable by Western blot. A KCTD1 crystal structure demonstrates that H33 is tightly packed in the protein (52). Accordingly the H33P mutation was suggested to induce destabilizing effects *via* significant steric clash with other residues (53). Mutations at the other BTB residues (A30E, H33P, G62D, and H74P) were found to impact thermostability and lead to aggregate-like formation (53), which likely leads to the increased accumulation we observed in transfected cells. In regard to effects on functionality, previous studies in cultured cells demonstrate that KCTD1 binds to AP-2α (10). These results have also been corroborated by binding studies with purified proteins (53). The BTB domain mutations reduce KCTD1’s ability to interact with AP-2α in both transfected cells and as recombinant proteins *in vitro* (8, 53). A recent structure suggests that the pathogenic mutations in KCTD1 support crucial folding of the BTB domain, which is required for pentameric assembly (9). Knockout of KCTD1 increases β-Catenin, whereas overexpression has the opposite effect (6, 7). This brings into question how AP-2α in cell culture can abrogate output of the Wnt pathway (10) while KCTD1 simultaneously inhibits both AP-2α and Wnt signaling (8, 9, 10, 11). Indeed recently *in vivo*, renal collecting duct selective AP-2α knockout did not alter total or phosphorylated (active) β-Catenin in mouse kidney lysates (54), suggesting nonredundant roles for KCTD1 and AP-2α in the Wnt signaling. We interpret the collective findings to support the plurality of cellular functions for KCTD1. The BTB domain mutations in KCTD1 serve as loss of function toward interplay with AP-2α. Separately, KCTD1 mutations increase their abundance, albeit in putative aggregate form, which maintains strong inhibition toward Wnt, Hh, and Notch cascades. In this connection, our study provides evidence that pathogenic BTB mutations also suppress the Hh pathway. Furthermore, we report that KCTD1 and the BTB mutants inhibit intracellular Notch signal transmission at the level of RBPJ regulation. In the Wnt pathway, our assay revealed that WT and BTB mutants reduced signaling by the same extent (except P31R). Yet in Hh assays, the BTB mutants reduced signaling even further compared with WT (except P31R). The same was found for the Notch assay except A30E had similar effect as KCTD1 WT. Given their increased abundance, the data suggest that further increasing KCTD1 level leads to enhanced inhibition of signaling at Hh and Notch pathways.

Our previous profiling revealed that KCTD1 can pull down with Gβγ in transfected cells, but the interaction is not increased following D2R→Gαo activation (21). Here, we demonstrate that KCTD1 does not interact with Gβγ following D1R (Gαs) or M3R (Gαq) stimulation nor does KCTD1 interfere with G protein activation. Seemingly, KCTD1 has no direct role in GPCR signaling. Yet KCTD1, and its stable BTB-domain mutants, clearly upregulate AC5. This sets up enhanced cAMP production as measured by directly stimulating ACs with forskolin. AC5 is classically sensitized by the direct effect of binding Gβγ (40, 41, 43). Previous work has demonstrated that the Gβγ effect on AC5 can be rescued by a nanobody-based Gβγ scavenger (20). Our data postulate that KCTD1-mediated upregulation of AC5 not only enhances cAMP but also allows AC5 to compete with other effectors for the pool of Gβγ. Even though cells endogenously express significantly more G proteins than effectors (55), our data demonstrate that AC5 is able to prevent Gβγ from interacting with both high (GRK3) and low (GIRK4) affinity effectors. This enables KCTD1 to bias GPCR signals toward AC5 and potentially dampen responses at other signaling modalities. This is conceptually similar to the kinetics of GIRK activation by Gβγ, which is rapidly desensitized by KCTD12 (directly uncouples Gβγ) (17, 26, 56) or RGS-mediated hydrolysis of GTP on Gα (induces re-association of Gα with Gβγ) (57, 58). Whereas in our case, AC5 serves to impede signaling at other effectors.

Our comprehensive investigation strengthens understanding of the manifold roles of signaling shaped by KCTD1. The multiple signal modalities associated with KCTD1 highlight its emergence in distinct pathological processes. Our findings overall provide a foundation for future studies on the significant role of KCTD1 in biology.

## Experimental procedures

### Mouse models

Experiments utilizing mouse models were approved by guidelines set forth by Augusta University’s Institutional Animal Care and Use Committee in compliance with the National Institutes of Health guidelines. Animals were housed under standard conditions with continuous access to both food and water while being maintained under a 12 h light/dark cycle with consistent temperature. Our study utilized both male and female mice from the previously described *CAMPER* (C57BL/6-Gt(ROSA)26Sortm1(CAG-ECFP∗/Rapgef3/Venus∗)Kama/J) (RRID:IMSR_JAX:032205) strain (36). Genotyping was performed by PCR with DNA tissue samples.

### Molecular biology

The following plasmids were obtained from Addgene: 3P-Luc (#113862), ICN1-GFP (#17623), Gli1 (#84922), NFAT-Fluc (#214665), mVenus-N1 (#addgene), 3xNLS-mTurquoise2 (#98817), pcDNA3.1 (#128034), AAV-hSyn-Cre-P2A-dTomato (#107738), AAV2/5 (#104964), and AdDeltaF6 (#112867). GαoA was obtained from the cDNA Resource Center (#GNA0OA0000). The following constructs have been previously described: KCTD1-myc-flag (21), KCTD12-myc-flag (21), mCherry-KRas (36), flag-AC5 (41), AC5-Nluc (23), KCTD1-Nluc (21), KCTD1-mVenus (23), KCTD5-Nluc (21), Nb5 (20), and Nb17 (20). GIRK4-Nluc and Kir2.2-Nluc were gifts from Dr Roderick MacKinnon (49). D1R, D2R, AC5, and ^T^Epac^VV^ were gifts from Dr Kirill Martemyanov (33, 41, 59). Venus 156-239-Gβ1, Venus 1-155-Gγ2, Gαq, and M3R were gifts from Dr Nevin Lambert (48). GRK3ct and GRK3ct-Nluc were gifts from Dr Cesare Orlandi (60, 61). Nluc-PM was generated by insertion of the synthesized Nluc with C-terminal plasma membrane targeting domain of KRas (Twist Bioscience) into the pTwist expression vector at EcoRI and BamHI sites. Nluc-Cyto was generated by insertion of the synthesized Nluc (Twist Bioscience) into the pTwist expression vector at EcoRI and BamHI sites. Gαs was generated by insertion of the synthesized ORF (Twist Bioscience) into the pTwist expression vector at HindIII and BamHI sites. HA-RBPJ was generated by insertion of the synthesized ORF (Twist Bioscience) into the pTwist expression vector at HindIII and BamHI sites. ICN1-mVenus was generated by insertion of synthesized ICN1 ORF (Twist Bioscience) at the N terminus of the mVenus-N1 vector using KpnI and AgeI sites. KCTD1-myc-flag variants were generated by synthesizing the ORF for KCTD1 point mutations (A30E, P31R, H33P, G62D, and H74P; Twist Bioscience) and replacing WT KCTD1 in the KCTD1-myc-flag vector using EcoRI and NotI sites. Fluc-KCTD1-flag was generated by inserting the synthesized ORF into pTwist vector at EcoRI and BamHI sites. The following C-terminal Nluc-tagged constructs were made by synthesis of the human ORF (Twist Bioscience) and cloning into AC5-Nluc vector at HindIII and KpnI sites: CIR1, KAT2A, MAML1, RITA1, RUNX1, and SKIP. Nluc-RBPJ was generated by cloning codon optimized Nluc (Twist Bioscience) into HA-RBPJ at HindIII and KpnI sites. Nluc-ICN1-mVenus was generated by subcloning Nluc from Nluc-RBPJ into ICN1-mVenus using HindIII and KpnI sites. AAV-Cre-P2A-KCTD1-WT was generated by replacing dTomato on the AAV-Cre-P2A-dTomato with synthesized KCTD1 ORF (Twist Bioscience) using PspXI and HindIII sites. AAV-Cre-P2A-KCTD1-H74P was subsequently generated by replacing WT KCTD1 with synthesized H74P variant ORF (Twist Biosciences) using EcoRI and HindIII sites. All plasmids generated in this paper were confirmed by whole plasmid sequencing (Plasmidsaurus).

### Cell culture

Human embryonic kidney cells (HEK293; Takara Bio #632180) and NIH/3T3 embryonic mouse fibroblast cells (ATCC #CRL1658) were cultured in DMEM (Gibco #11995) plus Fetalgro EX (7.5%; RMBio #FGX-BBT), MEM non-essential amino acids (Gibco # 11140050), penicillin (100 units/ml), and streptomycin (100 μg/ml) (Gibco # 10378-016). Cells were maintained in a 37 °C humidified incubator with 5% CO2. Plasmid transfection was performed as previously described with polyethylenimine (Polysciences #23966-100) and OptiMEM (Gibco #11058021) on PDL (Gibco #A38904) coated dishes or glass coverslips (18). siRNA was obtained from Santa Cruz Biotechnology: Control (Catalog # sc-37007), *Kctd1* (Catalog # sc-75373), *Kctd15* (Catalog #sc-97204). siRNA was diluted to 10 μM and 10 μl transfected with Lipofectamine 2000 (ThermoFisher # 11668019) following the manufacturer’s protocol. For experiments with siRNA and 3P-Luc, all reagents were transfected with Lipofectamine 2000.

### Adeno-associated virus production

An established protocol was utilized to package AAV particles encoding AAV-Syn-Cre-P2A-dTomato, AAV-SynCre-P2A-KCTD1-WT, and AAV-Syn-Cre-P2A-KCTD1-H74P (62). HEK293 cells were transfected with the AAV of interest, AAV2/5, and pAdDeltaF6. AAV particles were chloroform-extracted, concentrated to ∼10^13^ GC/ml *via* PCR estimation, and frozen in aliquots at −80 °C.

### Western blotting

HEK293 cells were detached in PBS containing 5 mM EDTA, centrifuged at 5000 rcf for 5 min, and resuspended in ice-cold lysis buffer (PBS plus 150 mM NaCl, 1% Triton-X, and protease inhibitor (Thermo Fisher Scientific #A32955). Lysate was sonicated for 15 s at 30% power (FisherBrand #FB50110) then collected after centrifugation (12,500 rcf for 5 min at 4 °C) followed by protein concentration measurement with the Pierce 660 nm reagent (Thermo Fisher Scientific #22660). Lysates were diluted to same concentration in an SDS-based buffer and incubated at 37 °C for 15 min, and 10-20 μg of sample was resolved on SDS polyacrylamide gels, transferred to PVDF membranes, and slowly rocked at room temperature in 5% dry non-fat milk (LabScientific #M0841) in PBS containing 0.1% Tween-20 (PBST). Primary and secondary antibodies were sequentially incubated in 1% dry non-fat milk PBST with copious PBST washing steps following each antibody incubation. The following primary antibodies were used: anti-GAPDH (6C5) (Millipore Sigma #MAB374), anti-HA (2-2.2.14) (Invitrogen #26183), anti-GFP (Invitrogen #A6455), anti-Flag (Proteintech #66008-4-Ig), anti-β-Actin (Cell Signaling Technology #3700S), anti-KCTD1 (Abcepta #AP4955b-ev), and anti-KCTD15 (Proteintech #20128-1-AP). The following HRP-conjugated secondary antibodies were used: anti-rabbit (Jackson ImmunoResearch #211-032-171) and anti-mouse (Jackson ImmunoResearch #115-035-174). Membranes were then exposed to ECL reagent (ThermoFisher #34580 or Kindle Biosciences #R1002), and protein bands were visualized utilizing the KwikQuant Imager (Kindle Biosciences #D1001). ImageJ was used to quantify band intensity.

### Immunoprecipitation

75 μg of total lysate was mixed with 0.5 μg anti-GFP antibody (Santa Cruz #SC-9996) and 5 μl Dynabeads Protein G (Invitrogen #10003D) and diluted to a total volume of 500 μl with lysis buffer. Samples were rotated for 1 h at 4 °C, washed three times by rotating with 500 μl lysis buffer for 10 min each time, resuspended in 50 μl of an SDS-based buffer, and incubated for 15 min at 37 °C. Samples (20 μl) were then analyzed by Western blot as described above.

### Confocal imaging

HEK293 cells were grown on glass coverslips for transfection with 3X-NLS-mTurquoise2, ICN1-mVenus, and mCherry-KRas with either pcDNA or KCTD1-myc-flag (1:1:1:1 ratio). Cells were fixed with 4% PFA and then mounted on slides for imaging through a 20X objective on a Leica Stellaris confocal microscope with HyD photomultiplier tube (PMT) adjusted to capture appropriate emission wavelengths. mTurquoise2 was excited by an OPSL 405 nm laser line (430-520 nm PMT emission). A supercontinuum white light laser was tuned to 515 nm for mVenus excitation (520–585 nm PMT emission). The supercontinuum white light laser was tuned to 587 nm for mCherry excitation (592–750 nm PMT emission). All images were acquired with identical laser intensity, digital gain, pinhole size, and line averaging.

### FRET cAMP imaging of cells

HEK293 cells were grown on glass coverslips for transfection with ^T^Epac^VV^ and AC5 with either pcDNA or KCTD1 (1:1:1 ratio). Cells were perfused at ∼2.5 mL/min with an ambient temperature buffer consisting of (in mM): NaCl (125), KCl (2.5), CaCl2 (2), MgCl2 (2), NaH2PO4 (1.25), NaHCO3 (10), glucose (10), and Hepes (5). Forskolin (Cayman Chemical #11018) was bath applied at 1 μM. As previously described (21, 23), images were acquired at 10 s intervals through a 20X objective on a custom Olympus microscope. FRET donor was excited by white light illumination (CoolLED pE-400) filtered to 436 nm (Chroma ET436/20×). FRET donor (455–500 nm) and acceptor (500–600 nm) images were split (Cairn OptoSplit II; Chroma ET480/40m and T455lp) for simultaneous acquisition through a Hamamatsu sCMOS (ORCA-Flash 4.0). ImageJ was used to calculate FRET values from total cell intensity.

### Luminescence assays

HEK293 cells were cotransfected with 3P-Luc and pcDNA or KCTD1 at a 1:1 ratio (1:3 ratio in Fig. 2, *B*, *D* and *F*). For Wnt pathway, cells were treated overnight with 100 ng/ml Mouse Wnt-3a Recombinant Protein (Gibco #315-20-10UG). For assays with internal luminescence control, cells were cotransfected with cytosolic Nluc or Fluc. The assay was performed after detaching cells with PBS containing 5 mM EDTA, centrifugation at 700 rcf for 5 min, and resuspending in assay buffer (PBS supplemented with 0.5 mM MgCl2 and 0.1% glucose). Cell suspension was treated with Nano-Glo (Promega #N113A) and luminescence measured on a SPECTROstar Omega plate reader (BMG Labtech) in a 96-well white opaque plate. For Hh pathway, cells were cotransfected with Gli1 and harvested the following day in the same manner. Cell suspension was treated with Bright-Glo (Promega #E2610) followed by luminescence measurement. For Notch pathway, cells were cotransfected with ICN1-GFP. The cell culture media were centrifuged the next day at 5000 rcf for 3 min. Supernatant was mixed with coelenterazine (10 μM final concentration) (NanoLight Technology #303) followed by luminescence measurement. For AC5 luminescence assays, HEK293 cells were transfected with AC5-Nluc and NFAT-Fluc with either pcDNA or KCTD1 (1:1:1 ratio). The assay was performed after detaching cells with PBS containing 5 mM EDTA, centrifugation at 700 rcf for 5 min, and resuspending in assay buffer. Nluc was measured by treating cells with Nano-Glo, and Fluc was measured by treating cells with Bright-Glo before recording luminescence measurements. Smoothened agonist HCl (Selleck Chemical #S7779) was incubated with cells overnight at 2.5 μM final concentration. For assays with an internal luminescence control (*i.e.* Nluc or Fluc), data were normalized by dividing the raw luminescence for the signal readout by the raw luminescence for the internal control. For comparison between groups, normalized data for each individual experiment were then divided by the average of the control. All raw and normalized values are presented within the manuscript.

### IP-luminescence

Similar to Western blot, HEK293 cells were harvested for 15 s sonication at 30% power in PBS containing 1% Triton X-100, 150 mM NaCl, 5 mM MgCl2, and Pierce protease inhibitor (Thermo Scientific #A32955). Total lysate (TL) luminescence was measured in a 96-well opaque white-wall plate on a SPECTROstar Omega plate reader (BMG Labtech). Luminescence from Fluc was recorded from 20 μg of total lysate by adding 2× FireFly substrate: 10 mM DTT, 1 mM ATP, and 3 mg/ml D-luciferin (Gold Biotechnology #LUCK-100) in MgCl2 lysis buffer. Luminescence from Nluc was recorded from a separate well containing from 20 μg total lysate after addition of 2× Nano-Glo substrate (Promega #N1110) in lysis buffer. IP was performed by mixing 300 μg of total lysate with 10 μl Pierce Anti-DYKDDDDK Magnetic Agarose (ThermoFisher #A36797) for 2 h while rotating at 4 °C in a total volume of 500 μl. Agarose was washed three times by rotation at 4 °C in 500 μl lysis buffer for 10 min. Samples were then resuspended in 70 μl lysis buffer, and 25 μl used directly for each luminescence reading. IP ratio is reported as raw Fluc value divided by Nluc value.

### BRET assay

The day after transfection, cells were detached with PBS containing 5 mM EDTA, centrifuged at 700 rcf for 5 min, and resuspended in assay buffer (PBS supplemented with 0.5 mM MgCl2 and 0.1% glucose). Cell suspension was mixed with Nano-Glo in a 96-well white opaque plate for simultaneous Nluc (475 nm) and Venus (535 nm) recordings from dual PMTs on a SPECTROstar Omega plate reader (BMG Labtech) at 100 ms intervals. Agonist was injected at indicated time in the graphs. BRET ratio was calculated by dividing raw intensity of 535 nm by 475 nm, as widely reported (48, 63). For D2R signaling, HEK293 cells were transfected with D2R, GαoA, Gβγ-Venus (Venus 156-239-Gβ1 and Venus 1-155-Gγ2), and effector (AC5-Nluc, Nluc-PM, Nluc-Cyto, Kir2.2-Nluc, GRK3ct-Nluc, or GIRK4-Nluc) with either pcDNA or plasmid of interest at a 1:2:1:1:4 ratio. For D1R signaling, HEK293 cells were transfected with D1R, Gαs, Gβγ-Venus (Venus 156-239-Gβ1 and Venus 1-155-Gγ2), and GRK3ct-Nluc with either pcDNA or KCTD1 at a 1:4:1:1:4 ratio. For M3R signaling, HEK293 cells were transfected with M3R, Gαq, Gβγ-Venus (Venus 156-239-Gβ1 and Venus 1-155-Gγ2), and GRK3ct-Nluc with either pcDNA or KCTD1 at a 1:2:1:1:4 ratio. Empty pcDNA vector was used to normalize DNA transfection ratios. Final concentration of agonist stimulation was 100 μM. Cells transfected with D1R or D2R were stimulated with dopamine, and cells transfected with M3R were stimulated with acetylcholine.

### Stereotaxic injection

Neonatal CAMPER pups (P0-P2) were ice-anesthetized and placed in a custom 3D-printed device (64) on a rodent stereotaxic frame (Kopf Instruments) with constant thermal anesthesia, as previously described (65). AAV particles were delivered to dorsal striatum (AP: +2.4 mm anterior to lambda, ML ± 1.0 mm, DV -1.7 mm) through a Hamilton syringe coupled to a 30-gauge needle. Bilateral injections were performed with 200 nl of AAV particles over a 4-min period followed by allowing the needle to remain in place for an additional minute before slow removal. Pups were then placed in a 33 °C incubator for 15 to 30 min to recover before returning to their home cage.

### Acute brain slices

As similarly described (36, 66), mice between 6-8 weeks of age were anesthetized with isoflurane followed by decapitation for brain extraction to mount in agarose and submerge in ice-cold oxygenated buffer containing (in mM): KCl (2.5), NMDG (93), glucose (25), Hepes (20), sodium ascorbate (5), sodium pyruvate (3), thiourea (2), NaH2PO4 (1.2), CaCl2 (0.5), MgCl2 (10), and NaHCO3 (30). Three hundred micrometer coronal striatal slices were then cut on a vibratome (Precisionary VF-310-0Z). Tissue sections were then incubated at 34 °C for 1 h in an oxygenated recovery buffer consisting of (in mM): NaCl (126), KCl (2.5), CaCl2 (2), MgCl2 (2), NaHCO3 (18), NaH2PO4 (1.2), and glucose (10). The sections were then transferred to an ambient temperature oxygenated recording buffer for imaging experiments that consisted of (in mM) NaCl (125), KCl (2.5), CaCl2 (2), MgCl2 (2), NaH2PO4 (1.25), NaHCO3 (25), and glucose (25).

### *2-*Photon FRET cAMP imaging

Brain slices were imaged in a recording chamber (Warner Instruments) while perfused with buffer (described above) at approximately 2 ml/min. FRET was performed by donor fluorophore excitation from a Ti:Sapphire laser (Coherent Chameleon Vision S) tuned to 850 nm paired with simultaneous acquisition of donor (455-500 nm) and acceptor (526-571 nm) fluorophores *via* dual photomultiplier tubes on a Zeiss 780 multiphoton confocal microscope imaged through a 20X W Plan-Apochromat objective. Forskolin was bath applied at 10 μM, and XYZ image stacks were acquired at 15 s intervals. Evoked dopamine was achieved by acquiring a single Z plane at 500 ms intervals and providing electrical stimulation from a single 10 ms pulse (650 μA) through a tungsten microelectrode (World Precision Instruments) inserted adjacent to the field of view. Stimulation was controlled by Pulser (Prizmatix) software and a DS3 isolated current stimulator (Digitimer) (67). FRET values were calculated from the intensity of the neuron cell body utilizing ImageJ tools. Directionality of responses to evoked dopamine was utilized to classify responses from D1R (*i.e.,* increased cAMP response), as previously described (20, 23).

### Statistical analysis

Unless indicated, all data represented as mean ± the standard error of the mean (SEM). GraphPad Prism 10 was utilized for analysis, as indicated in each appropriate figure legend. The numbers of biological replicates are also included in the figure legend with a minimum of three for each experiment.

## Data availability

All data are contained within the manuscript and supporting information.

## Supporting information

This article contains supporting information.

## Conflict of interest

The authors declare that they have no conflicts of interest with the contents of this article.
