# Supplementary material for: Pathogenic variants in KCTD1 disrupt cAMP signaling and cellular communication associated with developmental pathways
Source: J Biol Chem. 2025 Oct 12;301(12):110813. doi: 10.1016/j.jbc.2025.110813 (PMC12657730; doi:10.1016/j.jbc.2025.110813)
Supplement: Supplemental Figures [file mmc1.pdf]

Supplemental Figure 1

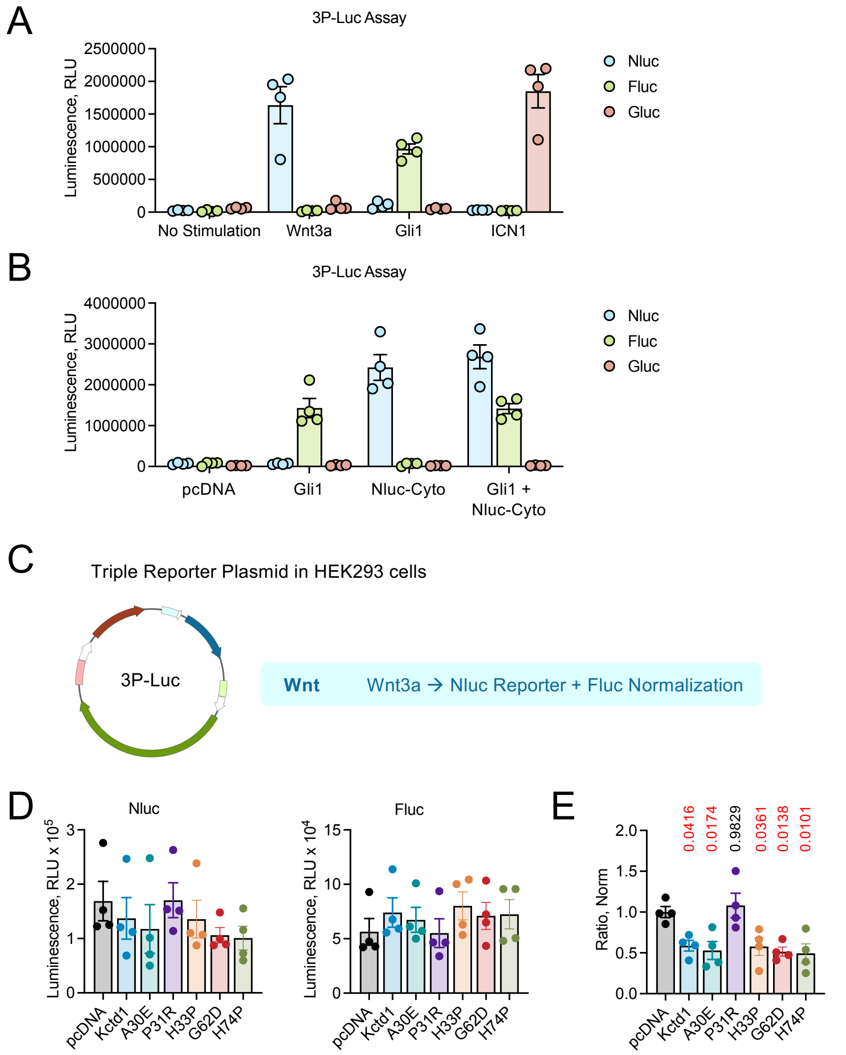

Figure S1: Impact of KCTD1 overexpression on 3P-Luc Wnt assay in HEK293 cells.

A. Luminescence from HEK293 cells transfected with 3P-Luc and stimulation by either Wnt3a peptide (100 ng/ml overnight), Gli1 co-transfection, or ICN1 co-transfection. Total transfection DNA was normalized using pcDNA empty vector. Luminescence was measured in each sample using Nano-Glo substrate (Nluc), Bright-Glo substrate (Fluc), and Coelenterazine substrate (Gluc). n= 4 independent experiments.

B. Luminescence from HEK293 cells co-transfected with 3P-Luc and either pcDNA, Gli1, Nluc-Cyto, or Gli1 and Nluc-Cyto. Total transfection DNA was normalized using pcDNA empty

vector. Luminescence was measured in each sample using Nano-Glo substrate (Nluc), Bright-Glo substrate (Fluc), and Coelenterazine substrate (Gluc). n= 4 independent experiments.

C. Schematic of 3P-Luc assay to examine Wnt (Nluc) signaling with cytosolic Fluc co-transfection to normalize data.

D. Luminescence quantification (Nluc and Fluc) for HEK293 cells co-transfected with 3P-Luc along with KCTD1 or pcDNA (1.0 μg each) followed by overnight treatment with 100 ng/ml Wnt3a. n= 4 independent experiments.

E. Quantification of luminescence ratio (Nluc Wnt signal divided by Fluc internal control) normalized to pcDNA control group. n= 4 independent experiments. One-way ANOVA, Dunnett posttest, multiple comparison to pcDNA group; exact p values indicated on the bar graph. All data represented as mean ± the standard error of the mean (SEM).

Supplemental Figure 2

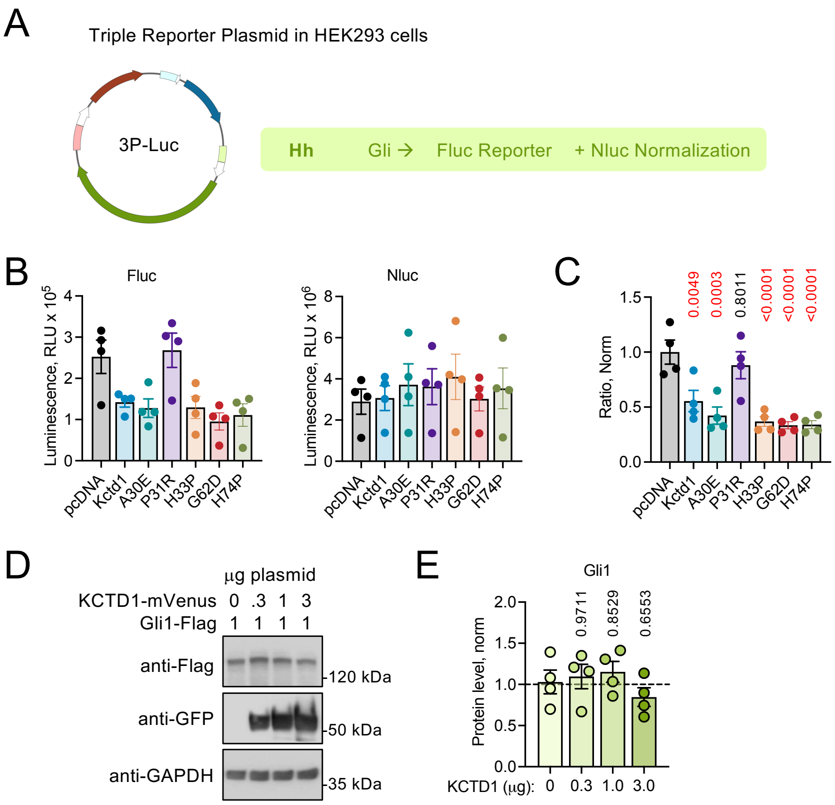

Figure S2: Impact of KCTD1 overexpression on 3P-Luc Hh assay in HEK293 cells.

A. Schematic of 3P-Luc assay to examine Hh (Fluc) signaling with cytosolic Nluc co-transfection to normalize data.

B. Luminescence quantification (Fluc and Nluc) for HEK293 cells co-transfected with 3P-Luc and Gli1 along with KCTD1 or pcDNA (1.0 µg each). n= 4 independent experiments.

C. Quantification of luminescence ratio (Fluc Hh signal divided by Nluc internal control) normalized to pcDNA control group. n= 4 independent experiments. One-way ANOVA, Dunnett posttest, multiple comparison to pcDNA group; exact *p* values indicated on the bar graph. All data represented as mean ± the standard error of the mean (SEM).

D. Western blot of Gli1-flag abundance with increasing KCTD1 transfection. Representative blot from four experiments.

E. Western blot quantification of Gli-flag level, normalized to 0 μg KCTD1-mVenus. n=4 independent experiments. One-way ANOVA, Dunnett posttest, multiple comparison to 0 μg KCTD1-mVenus group; exact *p* values indicated on the bar graph. All data represented as mean ± the standard error of the mean (SEM).

Supplemental Figure 3

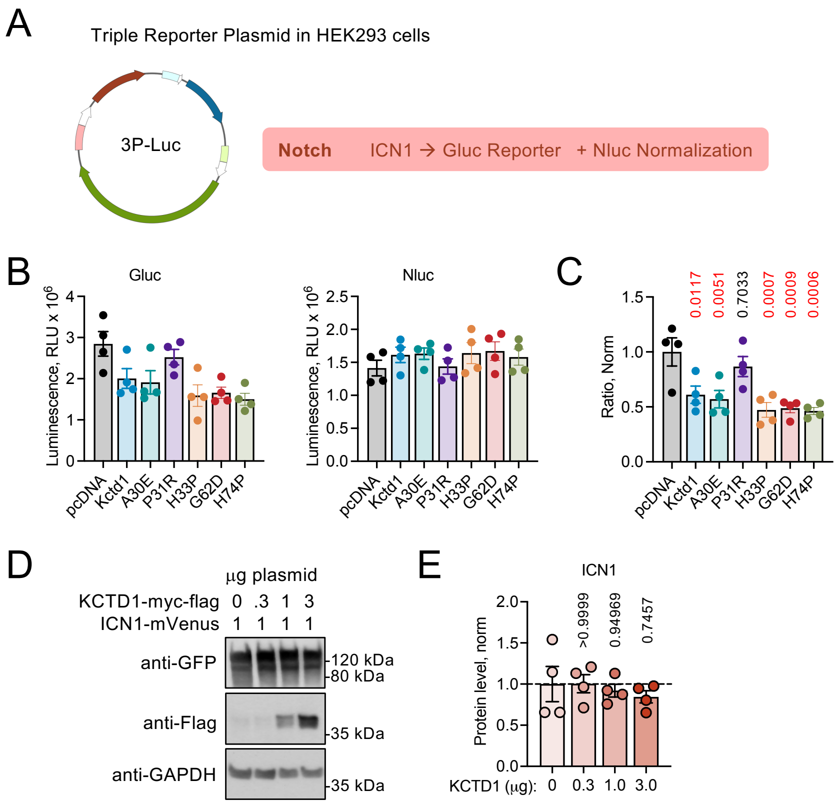

Figure S3: Impact of KCTD1 overexpression on 3P-Luc Notch assay in HEK293 cells.

A. Schematic of 3P-Luc assay to examine Notch (Gluc) signaling with cytosolic Nluc co-transfection to normalize data.

B. Luminescence quantification (Gluc and Nluc) for HEK293 cells co-transfected with 3P-Luc and ICN1 along with KCTD1 or pcDNA (1.0 μg each). n= 4 independent experiments.

C. Quantification of luminescence ratio (Gluc Notch signal divided by Nluc internal control) normalized to pcDNA control group. n= 4 independent experiments. One-way ANOVA, Dunnett posttest, multiple comparison to pcDNA group; exact *p* values indicated on the bar graph. All data represented as mean ± the standard error of the mean (SEM).

D. Western blot of ICN1-mVenus abundance with increasing KCTD1 transfection. Representative blot from four experiments.

E. Western blot quantification of ICN1-mVenus level, normalized to 0 μg KCTD1-flag. n=4 independent experiments. One-way ANOVA, Dunnett posttest, multiple comparison to 0 μg KCTD1-mVenus group; exact *p* values indicated on the bar graph. All data represented as mean ± the standard error of the mean (SEM).

Supplemental Figure 4

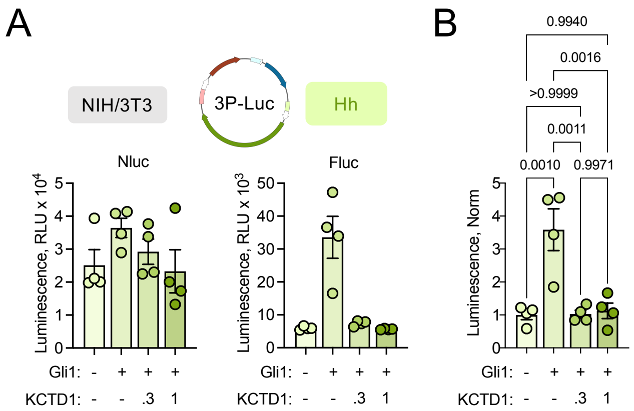

Figure S4: KCTD1 overexpression on 3P-Luc Hh assay in NIH/3T3 cells.

A. Luminescence quantification (Fluc and Nluc) for NIH/3T3 cells co-transfected with 3P-Luc and Gli1 along with increasing amounts of KCTD1. n= 4 independent experiments. All data represented as mean ± the standard error of the mean (SEM).

B. Quantification of luminescence ratio (Fluc Hh signal divided by Nluc internal control) normalized to control group (0 Gli1, 0 KCTD1). n= 4 independent experiments. One-way ANOVA, Dunnett posttest, multiple comparison to control group; exact *p* values indicated on the bar graph. All data represented as mean ± the standard error of the mean (SEM).

Supplemental Figure 5

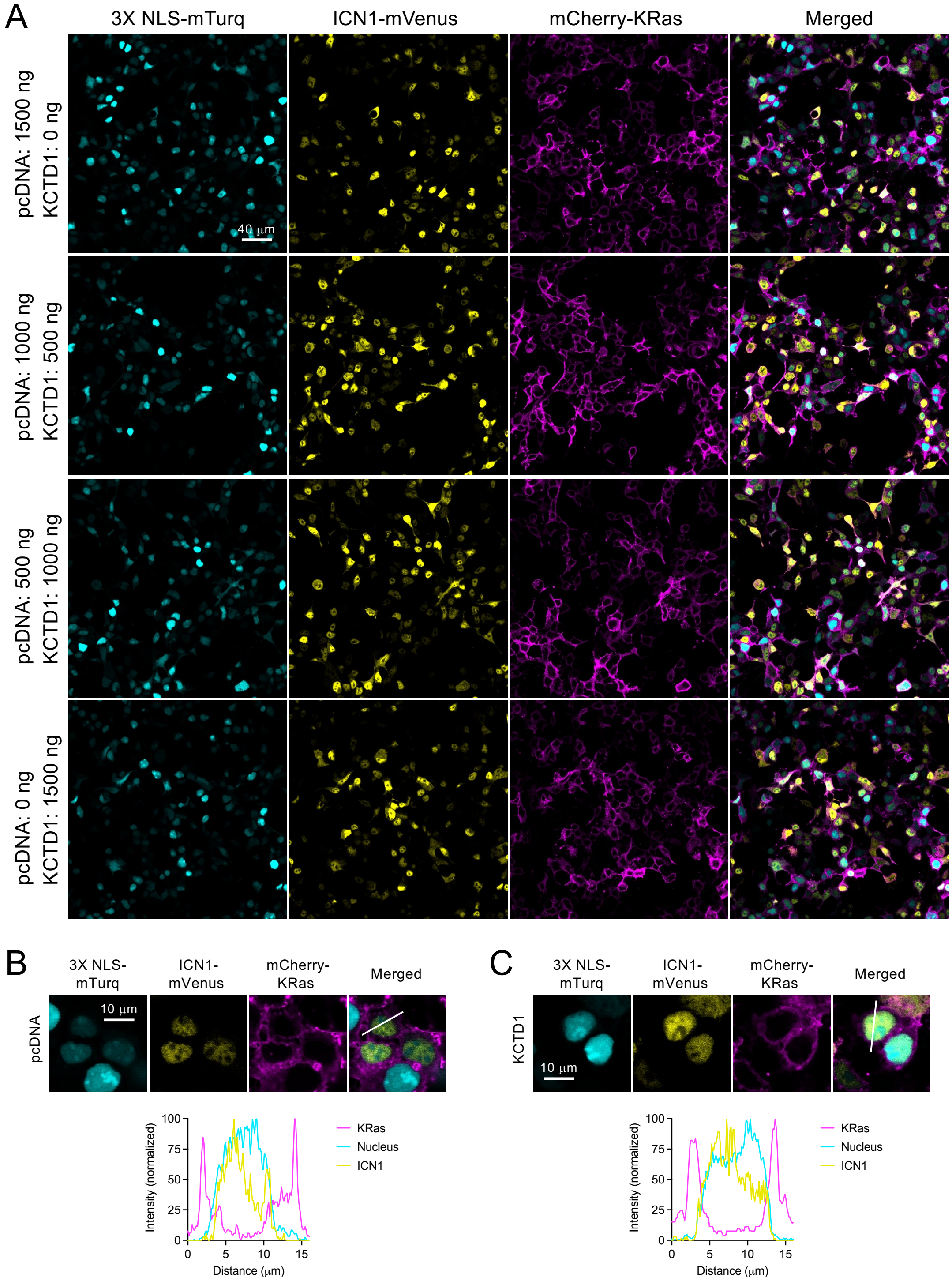

Figure S5: KCTD1 overexpression does not impact ICN1 localization in HEK293 cells.

A. Representative confocal images of HEK293 cells co-transfected with ICN1-mVenus, 3X NLS-mTurquoise2 (nuclear marker), mCherry-Kras (plasma membrane marker), and increasing amounts of WT KCTD1-myc-flag. White scale bar in merged image represents 10 μm. n=3 independent experiments.

B. Representative line scan intensity profile (normalized to max value for each fluorophore) for control transfection (1500 ng pcDNA, 0 ng KCTD1).

B. Representative line scan intensity profile (normalized to max value for each fluorophore) for KCTD1 overexpression (0 ng pcDNA, 1500 ng KCTD1).

Supplemental Figure 6

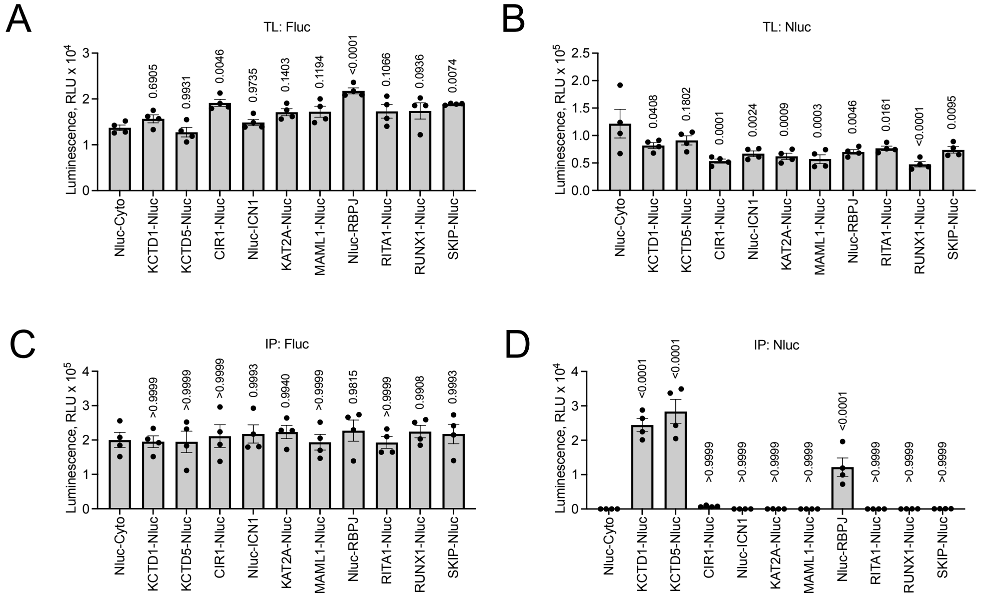

Figure S6: Raw luminescence values from Fluc-KCTD1-flag Co-IP in HEK293 cells.

A. Quantification of Fluc luminescence in the total lysate from co-transfection with Fluc-KCTD1-flag and Nluc tagged POI. n= 4 independent experiments.

B. Quantification of Nluc luminescence in the total lysate from co-transfection with Fluc-KCTD1-flag and Nluc tagged POI. n= 4 independent experiments.

C. Quantification of Fluc luminescence in the anti-flag pulldown from co-transfection with Fluc-KCTD1-flag and Nluc tagged POI. n= 4 independent experiments.

D. Quantification of Nluc luminescence in the anti-flag pulldown from co-transfection with Fluc-KCTD1-flag and Nluc tagged POI. n= 4 independent experiments.

One-way ANOVA, Dunnett posttest, multiple comparison to Nluc-Cyto group; exact p values indicated on the bar graph. All data represented as mean ± the standard error of the mean (SEM).

Supplemental Figure 7

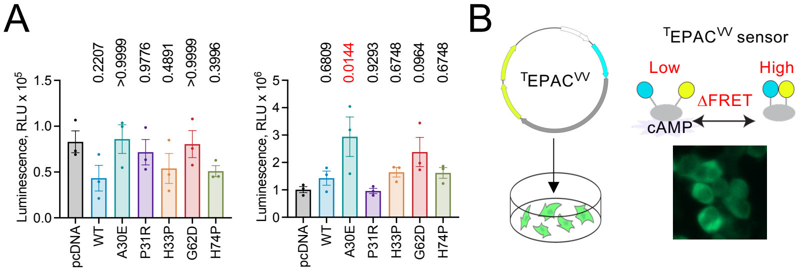

Figure S7: KCTD1 effect on AC5 level.

A. Quantification of Nluc and Fluc in HEK 293 cells co-transfected with AC5-Nluc and Fluc, respectively (1 μg). n=3 independent experiments. One-way ANOVA, Dunnett posttest, multiple comparison to pcDNA group.

B. Representative fluorescence image of HEK 293 cells transfected with the ^{T}Epac^{VV} biosensor.

Supplemental Figure 8

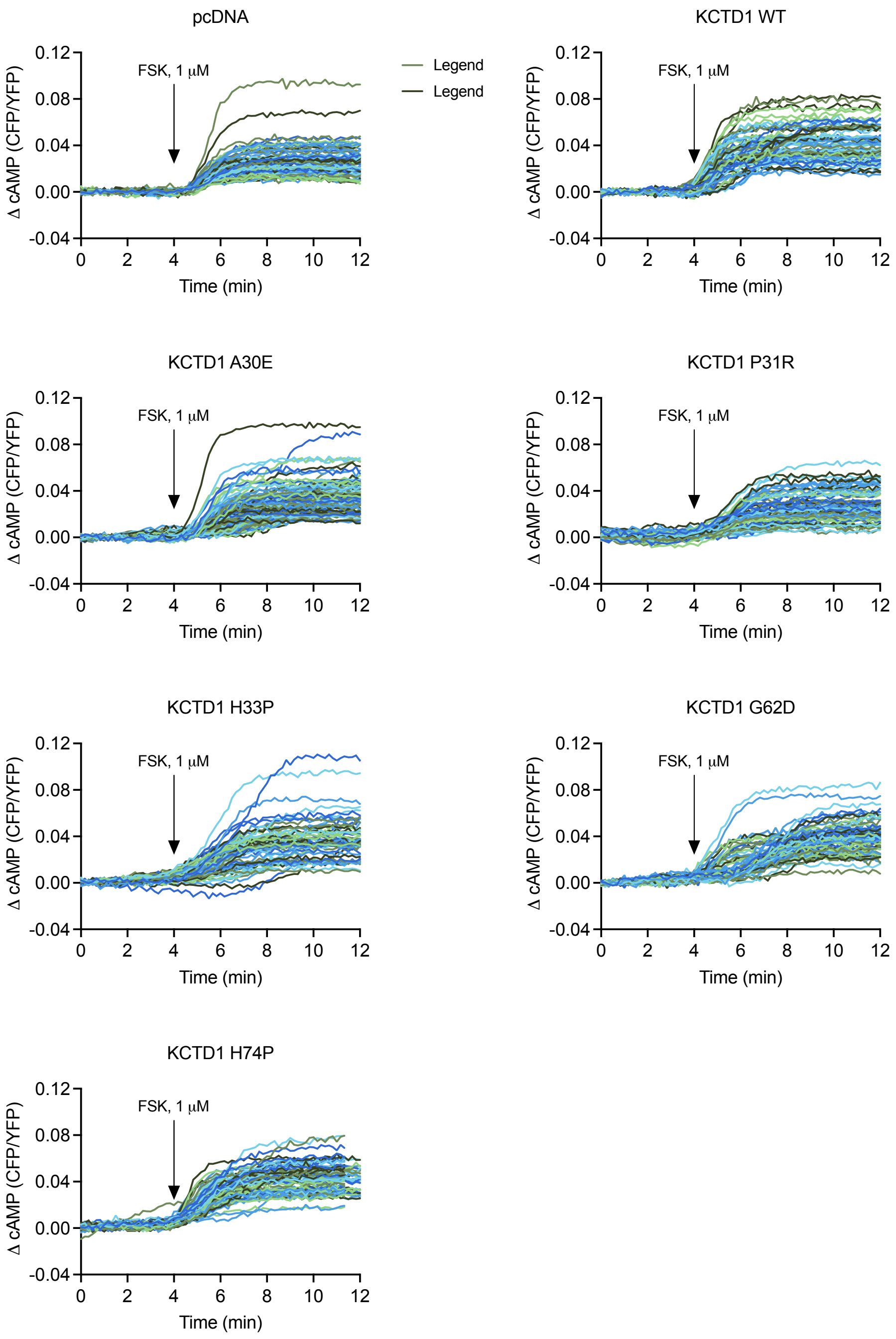

Figure S8: Individual ^{T}Epac^{VV}-transfected cell responses to forskolin stimulation.

All traces for individual cellular responses to 1 mM forskolin induced cAMP change in HEK293 cells co-transfected with ^{T}Epac^{VV} , Flag-AC5, and either pcDNA or KCTD1 (1 µg). Arrow indicates bath application of forskolin. Data collected from independent experiments with

following total number of cells recorded (in parenthesis) for each transfection condition: pcDNA (64), KCTD1 WT (64), KCTD1 A30E (86), KCTD1 P31R (72), KCTD1 H33P (57), KCTD1 G62D (71), KCTD H74P (70).

Supplemental Figure 9

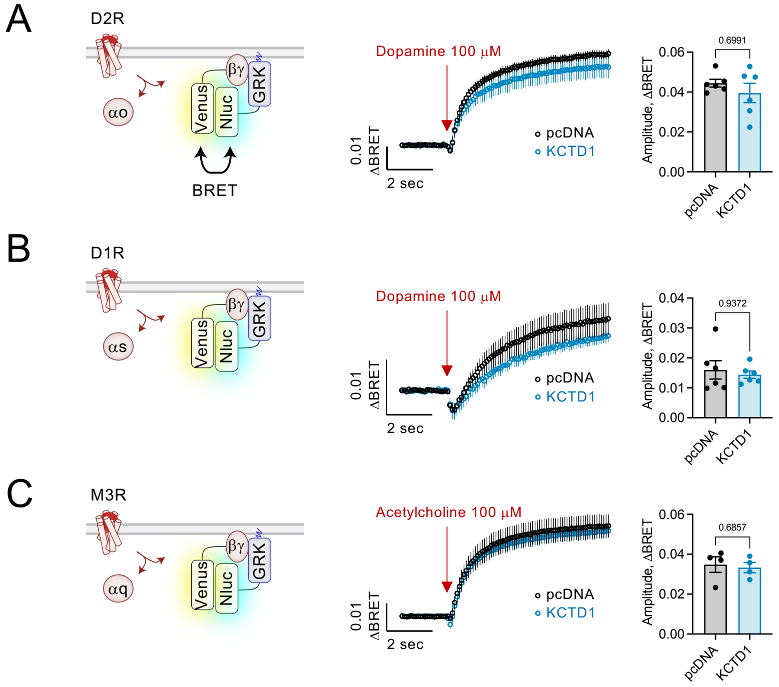

Figure S9: KCTD1 effect on Gβγ activation.

A. Average BRET trace and amplitude quantification induced by 100 μM dopamine from HEK293 cells co-transfected with D2R, Gαo, Gβγ-Venus, GRK3ct-Nluc, and either pcDNA or KCTD1. n=6 independent experiments, non-parametric test, Mann-Whitney U=15.

B. Average BRET trace and amplitude quantification induced by 100 μM dopamine from HEK293 cells co-transfected with D1R, Gαs, Gβγ-Venus, GRK3ct-Nluc, and either pcDNA or KCTD1. n=6 independent experiments, non-parametric test, Mann-Whitney U=17.

C. Average BRET trace and amplitude quantification induced by 100 μM acetylcholine from HEK293 cells co-transfected with M3R, Gαq, Gβγ-Venus, GRK3ct-Nluc, and either pcDNA or KCTD1. n=6 independent experiments, non-parametric test, Mann-Whitney U=6.

Supplemental Figure 10

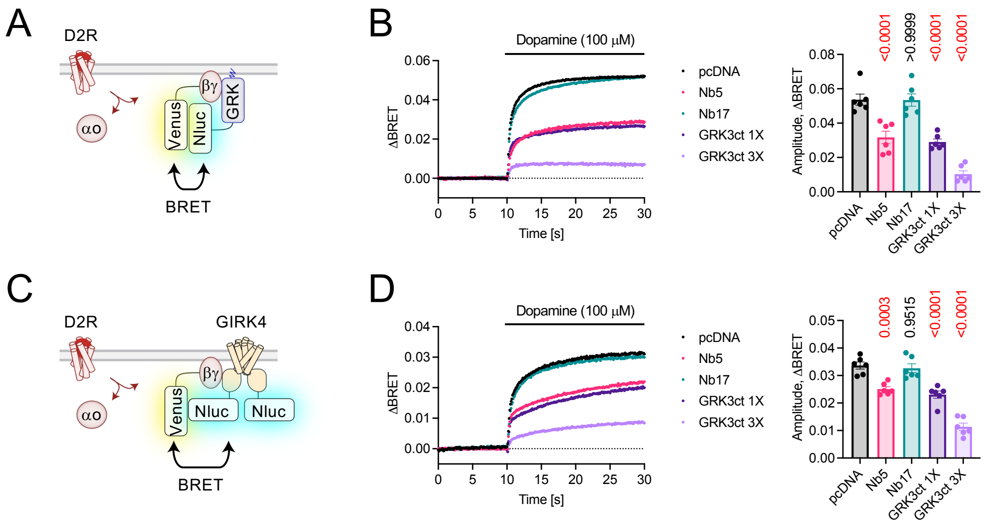

Figure S10: Effect of Gβγ scavengers on BRET signaling.

A. Schematic of BRET assay to monitor D2R signaling between Gβγ-Venus and GRK3ct-Nluc.

B. Average BRET trace and amplitude quantification induced by 100 μM dopamine from HEK293 cells co-transfected with D2R, Gαo, Gβγ-Venus, and GRK3ct-Nluc. n=6 independent experiments. One-way ANOVA, Dunnett posttest, multiple comparison to pcDNA group.

C. Schematic of BRET assay to monitor D2R signaling between Gβγ-Venus and GIRK4-Nluc.

D. Average BRET trace and amplitude quantification induced by 100 μM dopamine from HEK293 cells co-transfected with D2R, Gαo, Gβγ-Venus, and GIRK4-Nluc. n=6 independent experiments. One-way ANOVA, Dunnett posttest, multiple comparison to pcDNA group.
